# Supplementary material for: Chiral BINOL-phosphate assembled single hexagonal nanotube in aqueous solution for confined rearrangement acceleration
Source: Nat Commun. 2024 Mar 30;15:2799. doi: 10.1038/s41467-024-47150-6 (PMC10981660; doi:10.1038/s41467-024-47150-6)
Supplement: Supplementary file 1 — Supplementary Information [file 41467_2024_47150_MOESM1_ESM.pdf]

Supplementary Information

Chiral BINOL-Phosphate Assembled Single Hexagonal Nanotube in Aqueous Solution for Confined Rearrangement Acceleration

Kang Li*,[1,2,3] Wei-Min Qin,[1] Wen-Xia Su,[1] Jia-Min Hu,[1] and Yue-Peng Cai*[1,2,3]

[1] School of Chemistry, South China Normal University, Guangzhou 510006 (China)

[2] Guangzhou Key Laboratory of Energy Conversion and Energy Storage Materials, Guangzhou 510006 (China)

[3] The Joint Laboratory of Energy Materials Chemistry for SCNU and TINCI, Guangzhou 510006 (China)

* Corresponding Author

Email: likang5@m.scnu.edu.cn, caiyp@scnu.edu.cn

Table of Contents：

1. Self-assembly of Hexagonal Nanotubes and Characterization............................1

1.1 The procedure of self-assembling nanotubes in aqueous solution....................1
1.2 NMR characterization...1
1.3 HRESI-TOF-MS analysis..3
1.4 Single crystal X-ray structure determination..4
1.5 AFM testing...11
1.6 DOSY testing and estimation of the length of the nanotube..........................12
1.7 TEM testing...18

2. Catalytic Investigation..19

2.1 Synthesis of substrates...19
2.2 ITC titration between substrates and nanotube..21
2.3 General procedure for catalytic study..22
2.4 Reaction rate constant fitting and control experiment.................................22
2.5 Enthalpy of activation ($\Delta H^{\ddagger}$) and Entropy of activation ($\Delta S^{\ddagger}$) fitting.............29
2.6 NOESY measurement...32
2.7 Asymmetric catalysis investigation...33

3. Supplementary References..36

1. Self-assembly of Hexagonal Nanotubes and Characterization

1.1 The procedure of self-assembling nanotubes in aqueous solution

The R/S-HNOP (4-Hydroxy-2,6-di-2-naphthalenyl-4-oxide-dinaphtho[2,1-d:1',2'-f] [1,3,2] dioxaphosphepin) enantiomer (1mg, 0.0017 mmol) was dissolved in the DMSO (200 μL) and H_2O (400 μL) mixture containing equivalent amount of NaOH (0.0017 mmol). The resulting solution was stirred for 10 min at room temperature and stored for next investigation.

1.2 NMR characterization

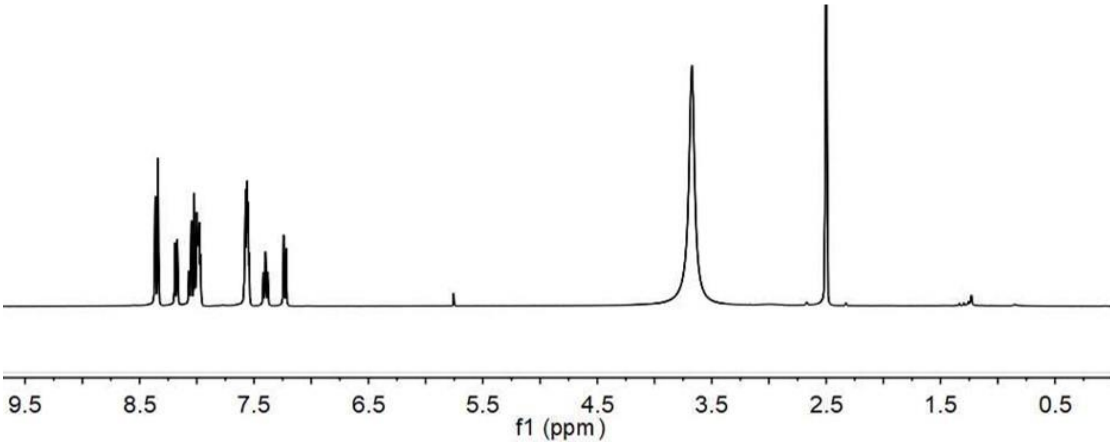

Supplementary Figure 1. ^{1}H NMR spectrum of deprotoned HNOP in DMSO-d_6.

^{1}H NMR (400 MHz, DMSO-d_6, 298 K): δ 8.36 (s, 1H), 8.33 (s, 1H), 8.18 (d, J = 8.8 Hz, 1H), 8.03 (m, 2H), 7.98 (m, 2H), 7.56 (m, 3H), 7.39 (t, J = 8.0 Hz, 1H), 7.23 (d, J = 8.4 Hz, 1H).

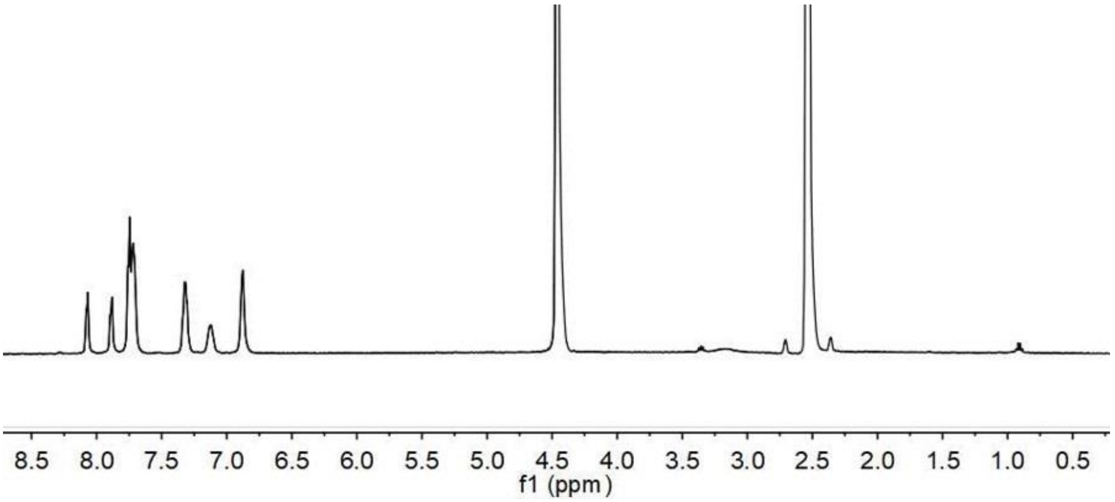

Supplementary Figure 2. ^{1}H NMR spectrum of assembled nanotube in DMSO-d_6/D_2O mixture (v/v = 1/2)

^{1}H NMR (400 MHz, DMSO-d_6/D_2O = 1/2, 298 K): δ 8.07 (br, 1H), 7.89 (br, 1H), 7.75 (m, 2H), 7.65-7.79 (br, 5H), 7.32 (m, 2H), 7.12 (br, 1H), 6.87 (br, 1H).

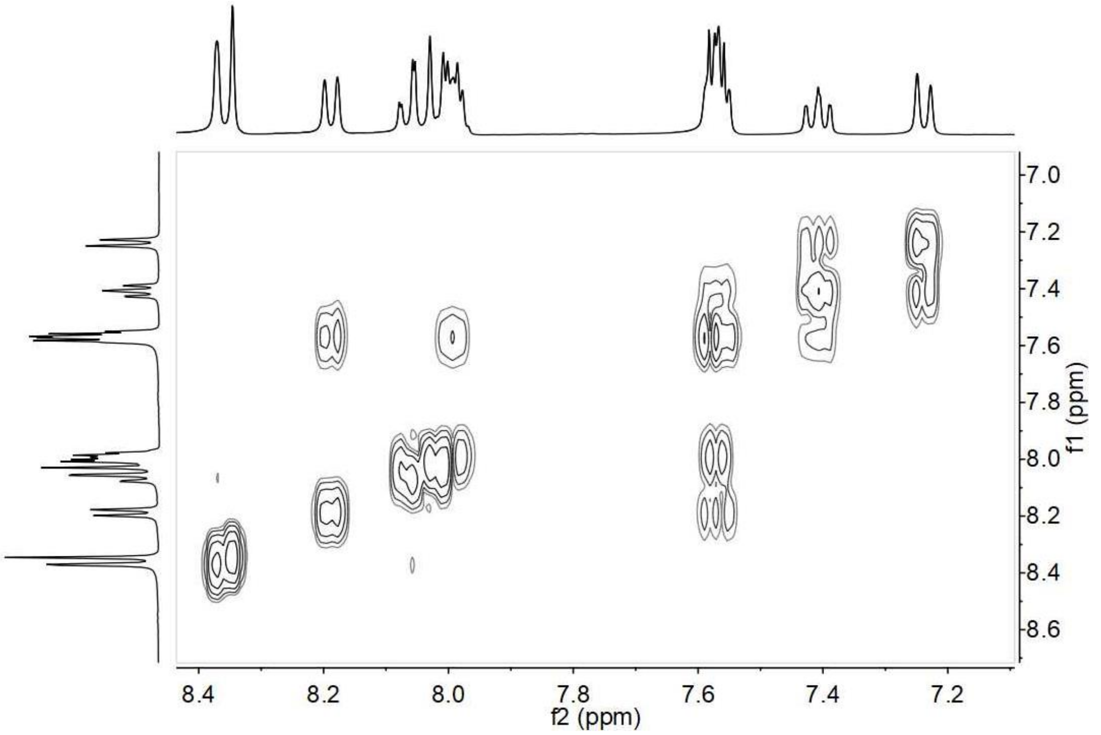

Supplementary Figure 3. ^{1}H-^{1}H COSY spectrum of deprotoned HNOP in DMSO-d_6 (400 MHz, 298 K).

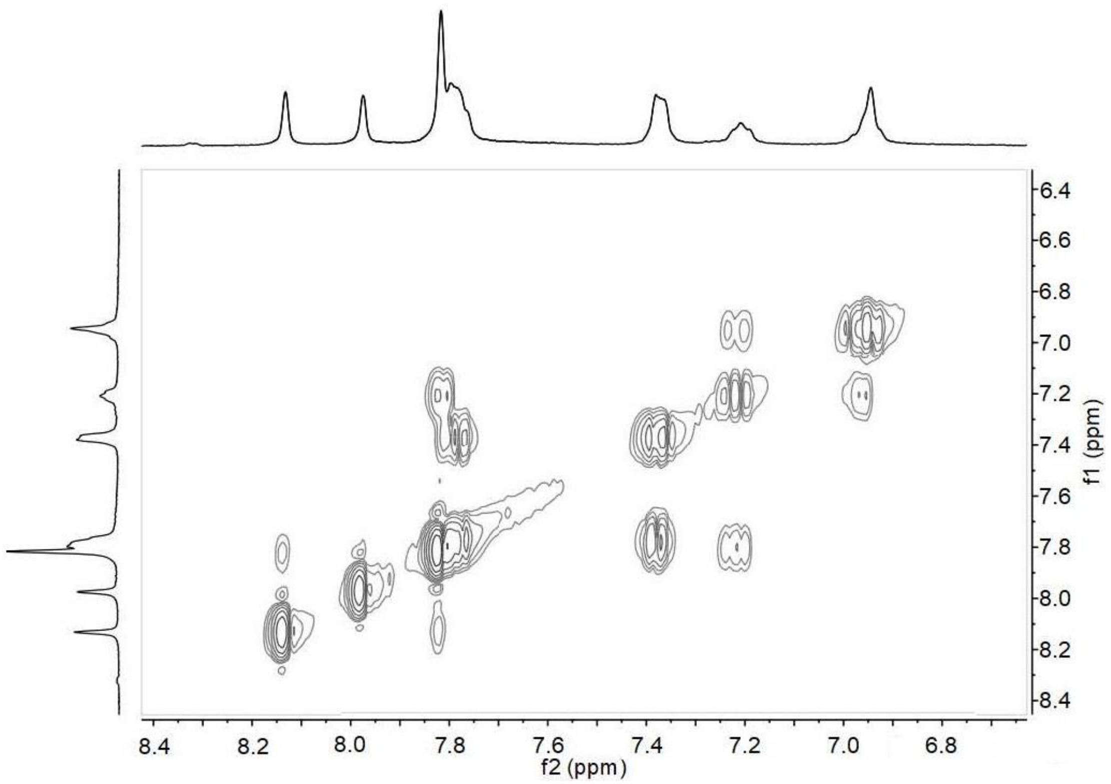

Supplementary Figure 4. ^1H-^1H COSY spectrum of assembled nanotube in DMSO-d_6/D_2O mixture (v/v = 1/2) (400 MHz, 298 K).

1.3 HRESI-TOF-MS analysis

(a)

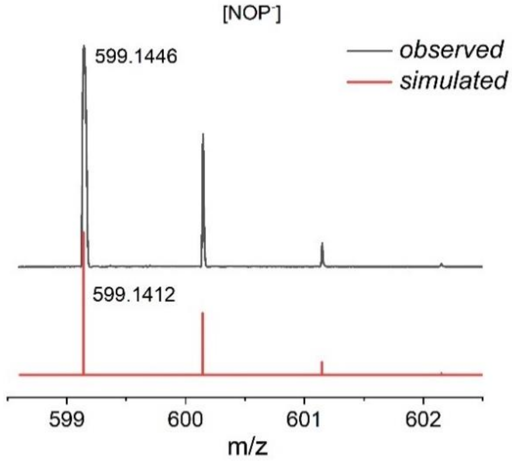

(b)

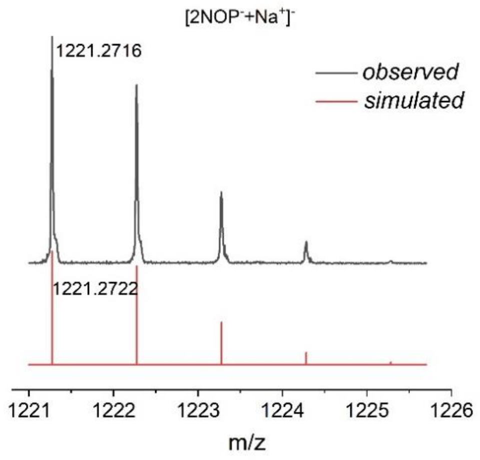

(c) (d)

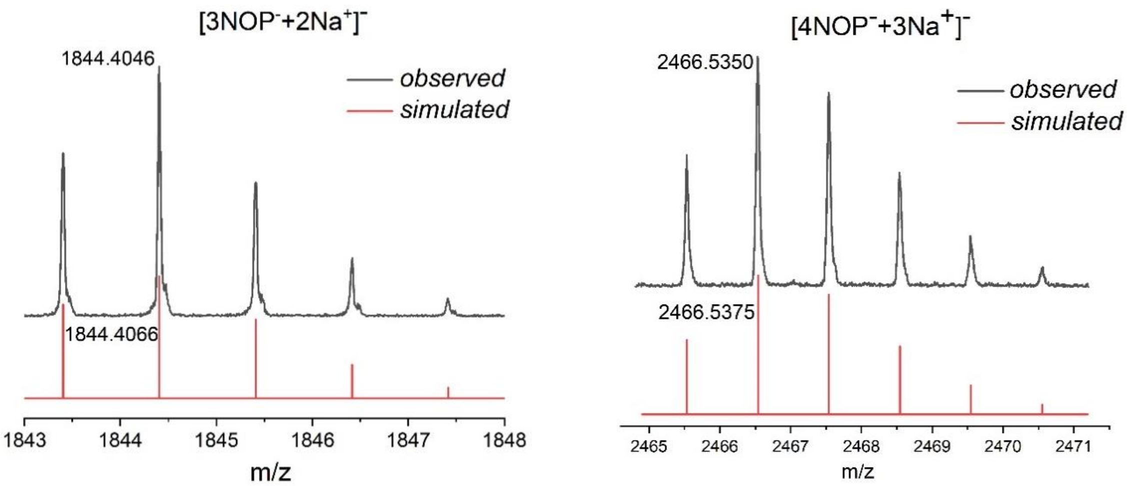

Supplementary Figure 5. (a-d) The experimental and assigned simulated isotopic distribution patterns ranging from 500-2500 (m/z).

1.4 Single crystal X-ray structure determination

Both single crystals of nanotube enantiomers suitable for X-ray diffraction were obtained from their corresponding aqueous solution after keeping for 5 days. Reflection intensity data were collected at 293 K on an Agilent SuperNova X-ray diffractometer using micro-focus X-ray sources (Cu-K_{α}, $\lambda = 1.54184$ Å). The crystals were kept at 293.8(5) K during data collection. Using Olex2,[1] the structures were solved with the SHELXT[2] structure solution program using Intrinsic Phasing and refined with the olex2.refine[3] refinement package using Levenberg-Marquardt minimisation. The hydrogen atoms were placed in geometrically calculated positions and included in the refinement process using riding model. Crystal and refinement parameters are listed in Table S1. The SQUEEZE method was used to remove all solvent molecules and Q peaks that located in the large cavity channels. Both structural cif files were deposited on the CCDC data set with deposition number of 2258255 (R-nanotube) and 2258256 (S-nanotube).

Supplementary Table 1. Crystal data and structure refinement parameters

**Identification code**	**R-nanotube**	**S-nanotube**
Empirical formula	$C_{40}H_{25}O_4P$	$C_{40}H_{25}O_4P$
Formula weight	600.616	600.616
Temperature/K	293.8(5)	293.9(4)
Crystal system	hexagonal	hexagonal
Space group	$P6_5$	$P6_1$
a/Å	25.1559(8)	25.1624(17)
b/Å	25.1559(8)	25.1624(17)
c/Å	10.8197(3)	10.8377(6)
α/°	90	90
β/°	90	90
γ/°	120	120
Volume/Å^3	5929.6(3)	5942.5(9)
Z	6	6
$\rho_{calc,}$ g/cm^3	1.009	1.309
μ/mm^{-1}	0.881	1.188
F(000)	1879.5	2472.0
Reflections collected	14020	26371
Independent reflections	7159 [R_{int} = 0.0354, R_{sigma} = 0.0442]	7360 [R_{int} = 0.0607, R_{sigma} = 0.0525]
Data/restraints/parameters	7159/3/411	7360/1/406
Goodness-of-fit on F^2	1.025	0.966
Final R indexes [I>=2σ (I)]	R_1 = 0.0919, wR_2 = 0.2432	R_1 = 0.0489, wR_2 = 0.1043
Final R indexes [all data]	R_1 = 0.1163, wR_2 = 0.2763	R_1 = 0.0875, wR_2 = 0.1226
Largest diff. peak/hole / e Å^{-3}	0.90/-0.32	0.11/-0.15
Flack parameter	0.01(5)	0.00(2)
CCDC Number	2258255	2258256

Supplementary Table 2. Selected bond lengths (Å) and bond angles (°) of R-nanotube.

P1-O1	1.612(4)	C19-C14	1.425(9)
P1-O2	1.615(4)	C19-C18	1.404(9)
P1-O3	1.481(5)	C11-C20	1.363(7)
P1-O4	1.466(6)	C11-C10	1.491(7)
O1-C21	1.398(6)	C11-C12	1.423(8)
O2-C1	1.381(6)	C1-C10	1.426(7)
P1-O1	1.612(4)	C19-C14	1.425(9)
P1-O2	1.615(4)	C19-C18	1.404(9)
C23-C22	1.428(7)	C10-C9	1.363(8)
C23-C24	1.412(7)	C33-C32	1.403(7)
O2-P1-O1	102.92(17)	C18-C19-C14	118.6(5)
O3-P1-O1	110.9(3)	C10-C11-C20	120.7(5)
O3-P1-O2	105.4(3)	C12-C11-C20	119.7(5)
O4-P1-O1	105.5(3)	C12-C11-C10	119.3(5)
O4-P1-O2	111.2(3)	C2-C1-O2	119.0(4)
O4-P1-O3	119.6(3)	C10-C1-O2	118.5(4)
C21-O1-P1	119.4(3)	C10-C1-C2	122.5(5)
C1-O2-P1	118.0(3)	C11-C20-C19	121.6(5)
C24-C23-C22	122.7(4)	C1-C10-C11	121.4(5)
C28-C23-C22	118.1(4)	C9-C10-C11	121.2(5)

Supplementary Table 3. Selected bond lengths (Å) and bond angles (°) of S-nanotube.

P1-O1	1.614(3)	C19-C14	1.405(7)
P1-O2	1.622(3)	C19-C18	1.404(7)
P1-O4	1.489(4)	C4-C5	1.377(6)
P1-O3	1.458(4)	C2-C1	1.369(6)
O1-C21	1.404(5)	C9-10	1.376(6)
O2-C1	1.392(5)	C24-C25	1.376(6)
C23-C22	1.436(6)	C10-C1	1.414(6)
C23-C28	1.416(6)	C33-C32	1.411(6)
C23-C24	1.411(6)	C33-C38	1.394(7)
C8-C41	1.432(6)	C33-C34	1.397(7)
O1-P1-O2	103.40(15)	C11-C20-C19	121.6(4)
O4-P1-O1	110.9(2)	C41-C2-C22	121.2(4)
O4-P1-O2	104.8(2)	C1-C2-C22	119.1(4)
O3-P1-O1	105.8(2)	C1-C2-C41	119.7(4)
O3-P1-O2	111.3(2)	C10-C9-C8	122.8(4)
O3-P1-O4	119.6(2)	C25-C24-C23	120.3(4)
C21-O1-P1	119.4(3)	C9-C10-C11	121.1(4)
C1-O2-P1	117.7(3)	C9-C10-C1	116.7(4)
C28-C23-C22	118.1(4)	C1-C10-C11	122.0(4)
C24-C23-C22	122.9(4)	O2-C1-C10	117.7(4)

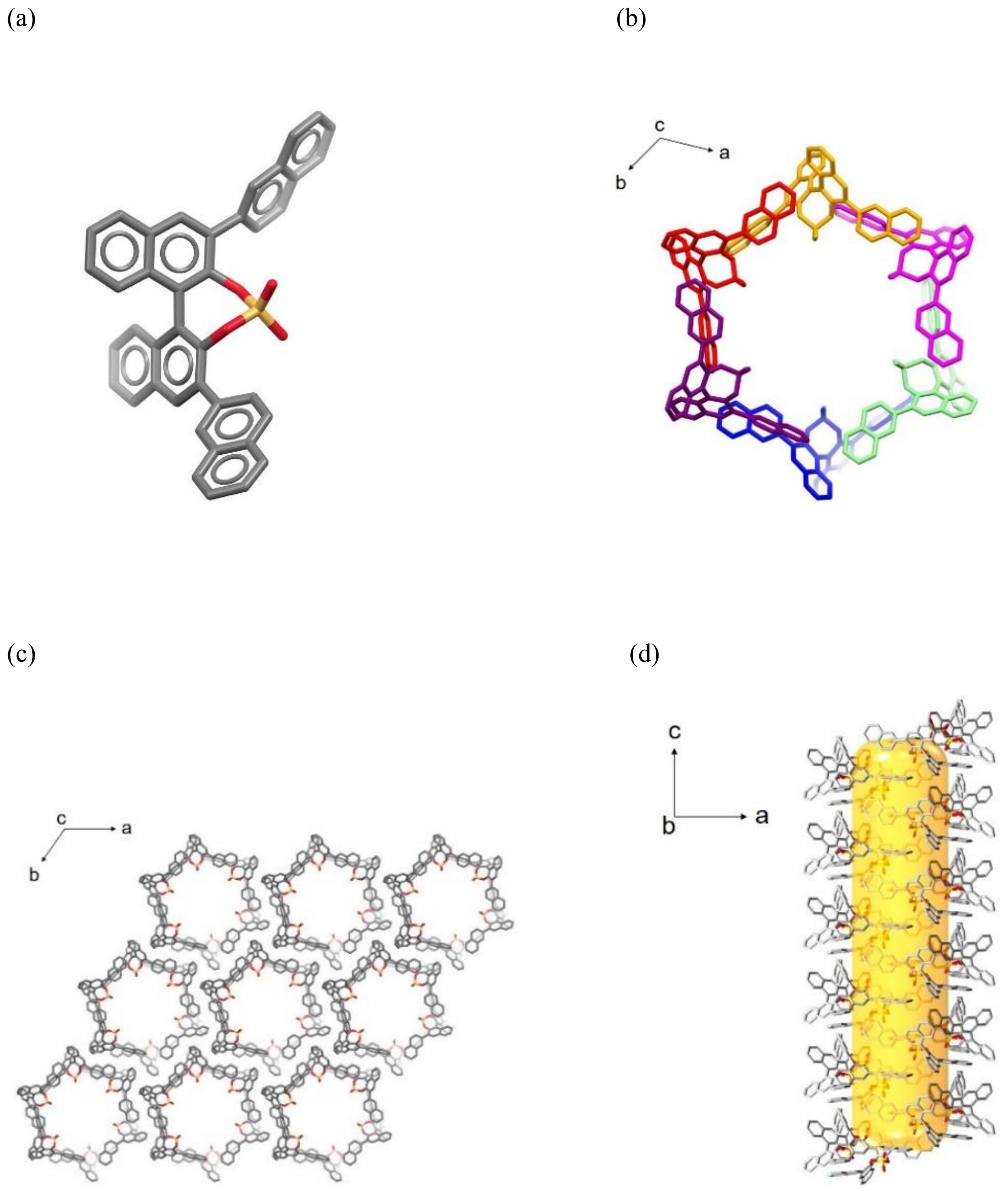
(a)
(b)
c
a
b
(c)
c
a
b
(d)
c
b
a

(e)

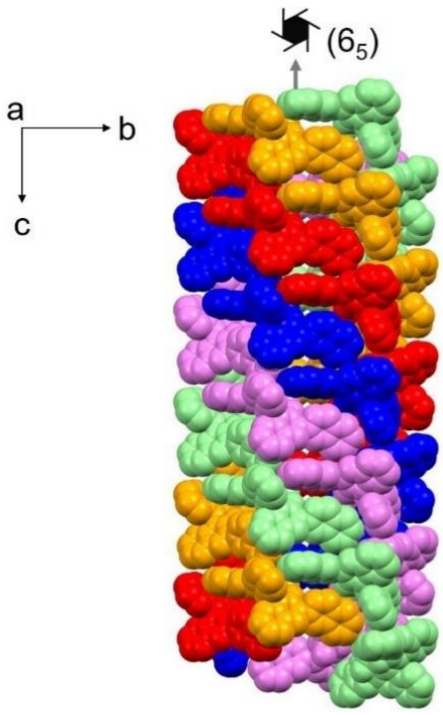

(f)

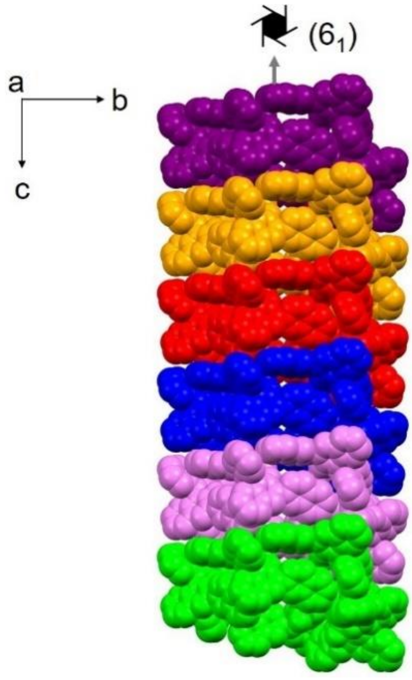

Supplementary Figure 6. (a) crystal structure of S-HNOP subcomponent; (b) six S-HNOP assembled hexagon; (c) S-nanotube packing along *c* axis; (d) single S-nanotube with interior channel cavity (yellow color); (e-f) space-filling mode shows the 6_5 and 6_1 screw axes across the centre of S-nanotube. (Hydrogen atom was omitted for clarity)

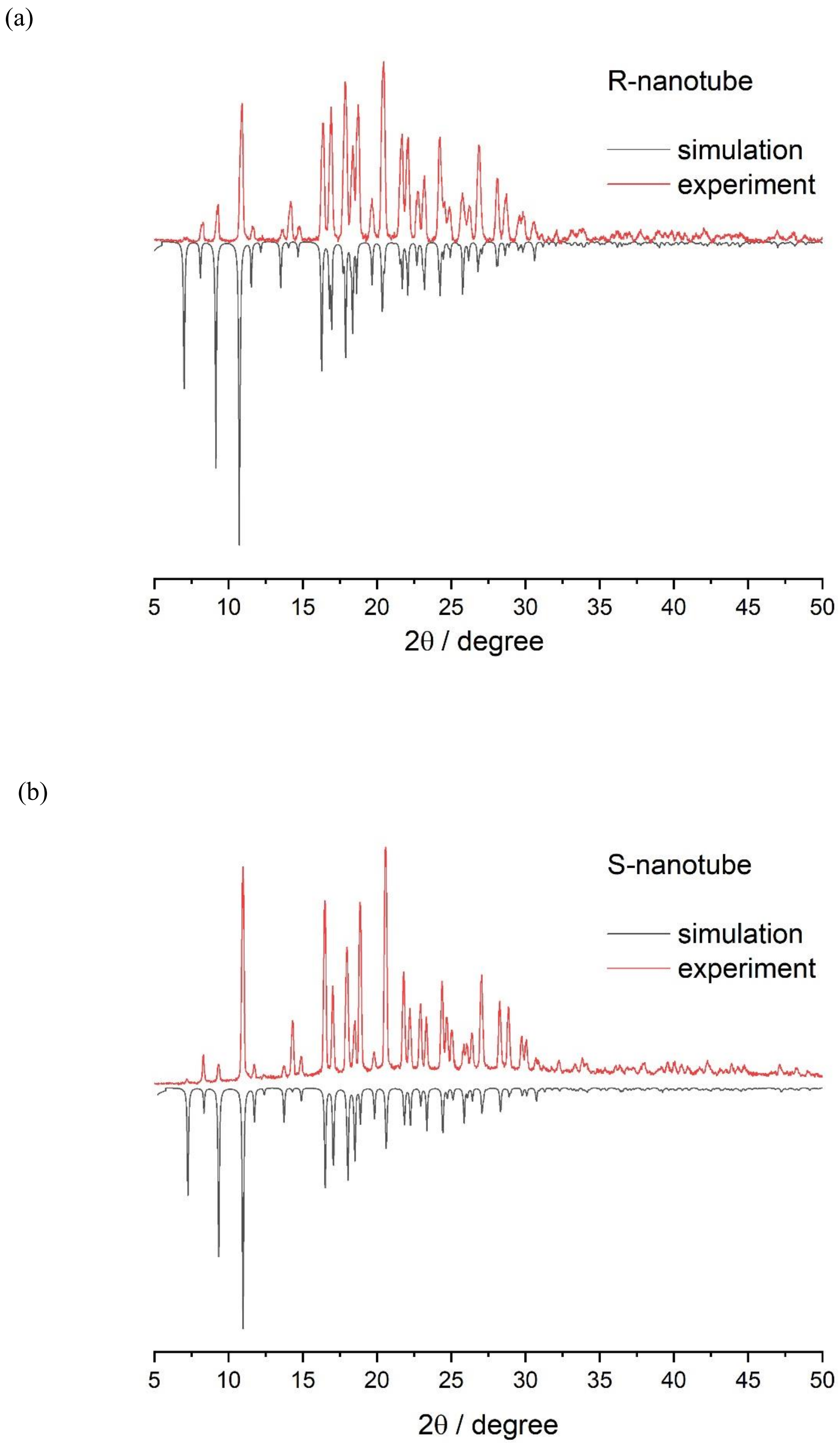

Supplementary Figure 7. (a-b) comparison of powder X-ray diffraction between experimental and simulated patterns for R-nanotube and S-nanotube.

1.5 AFM testing

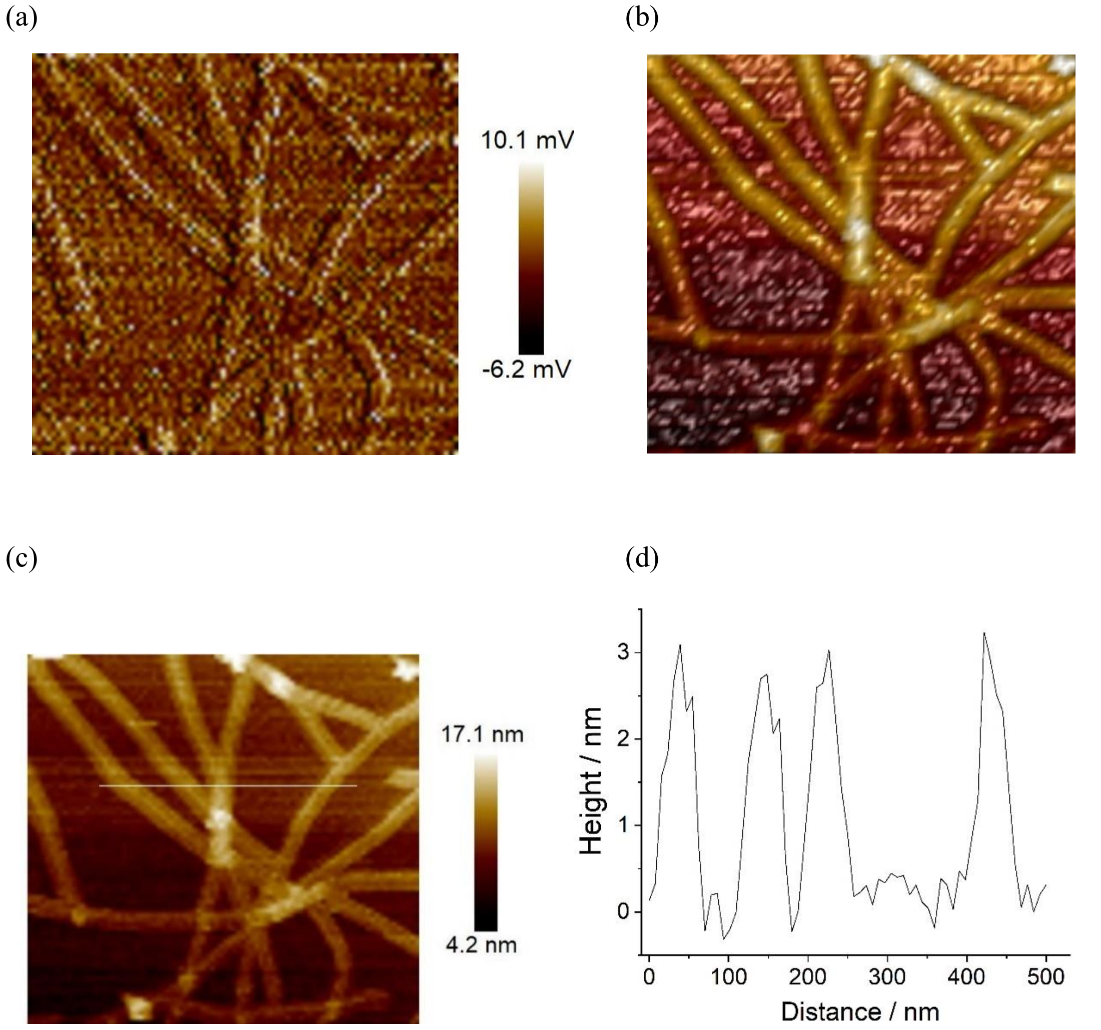

Supplementary Figure 8. (a) Pristine AFM images of aqueous solution of R-nanotube (15 mM) on mica; (b) 3D height profile of the corresponding R-nanotube; (c) 2D profile of R-nanotube and its corresponding height curve along the selected white line (d).

1.6 DOSY testing and estimation of the length of the nanotube

(a)

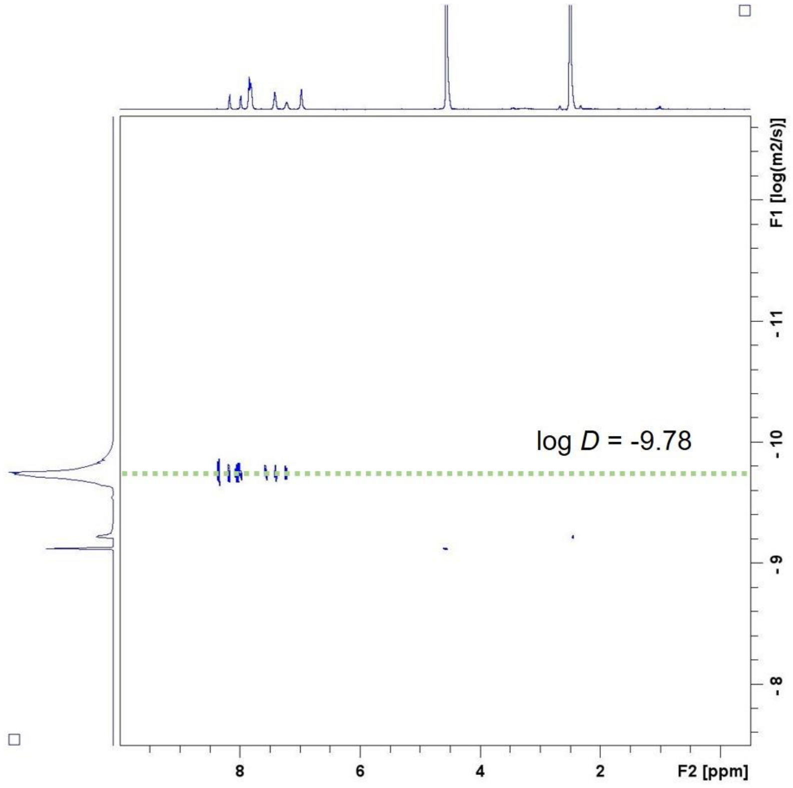

(b)

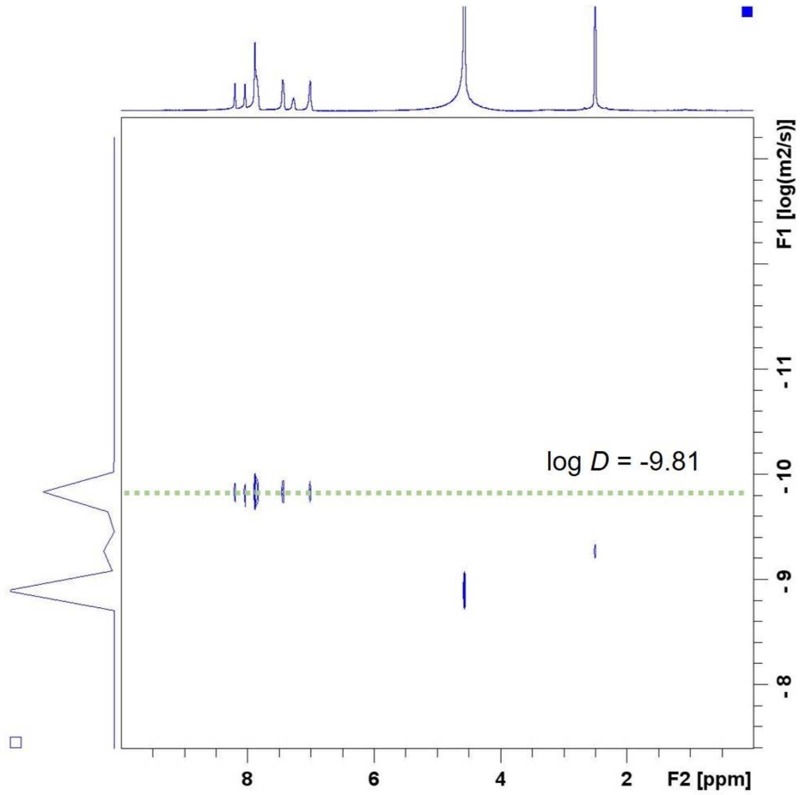

(c)

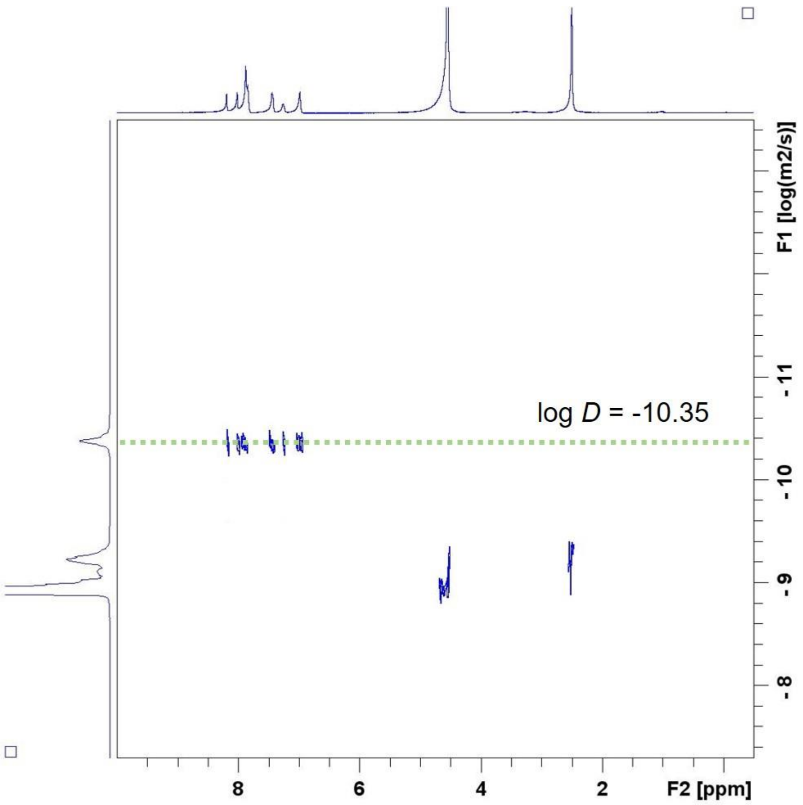
log *D* = -10.35
F1 [log(m2/s)]
-11
-10
-9
-8
8
6
4
2
F2 [ppm]

(d)

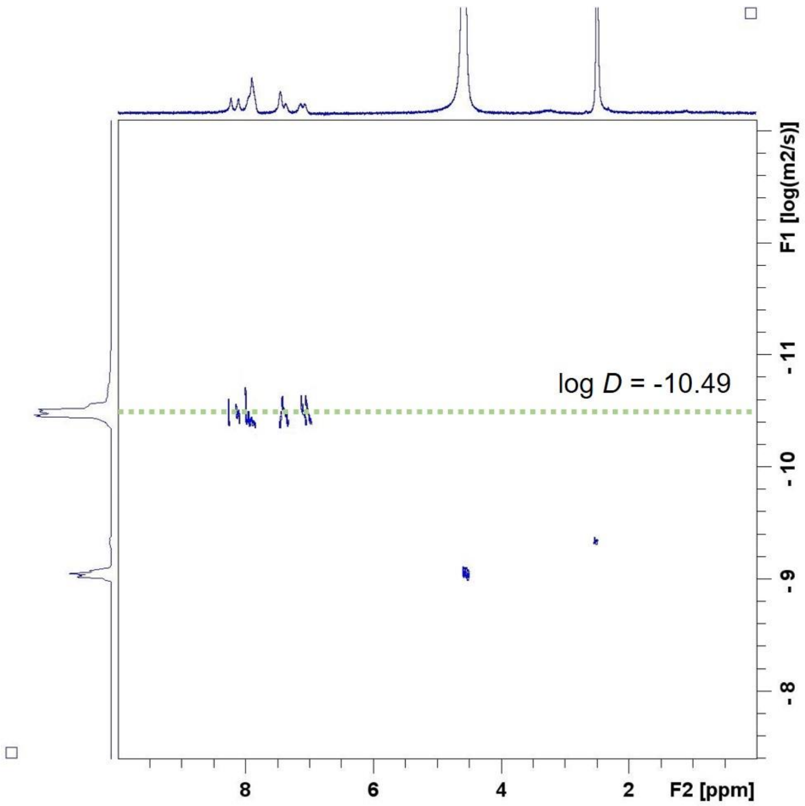
log *D* = -10.49
F1 [log(m2/s)]
-11
-10
-9
-8
8
6
4
2
F2 [ppm]

(e)

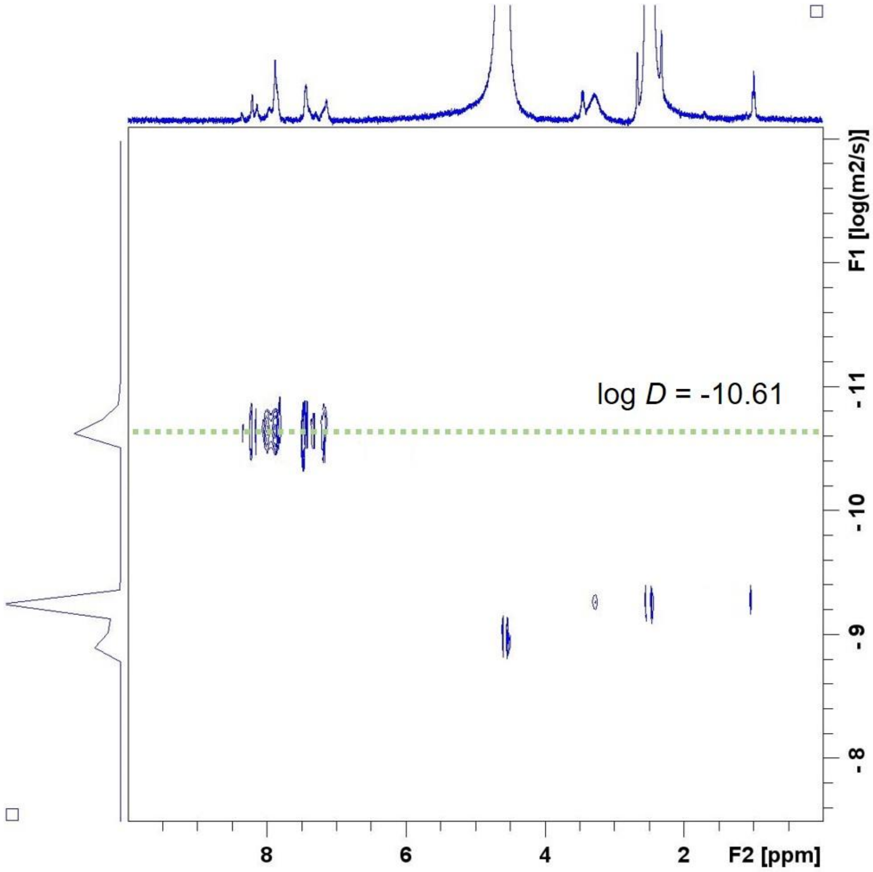

Supplementary Figure 9. Concentration-dependent DOSY spectra of Cat1 at (a) 2.8 mM, (b) 5.6 mM, (c) 8.4 mM, (d) 10.0 mM and (e) 12.0 mM. (400 MHz, DMSO-d_6/D_2O=1/2, 298 K)

Calculation of the length of the nanotube

Stokes-Einstein equation (cylinder model approximation)

$$D = \frac{kT}{3\pi\eta L}(\ln p + v) \quad \text{(Supplementary Equation 1)}$$

$$v = 0.312 + 0.565p^{-1} - 0.100p^{-2}$$

D: Diffusion coefficient

η: Viscosity of solvent

k: Boltzmann constant

T: Temperature

L: Length of cylinder

p: Axial ratio (*L*/*d*)

d: Diameter of cylinder

Note: This equation is valid under the condition of $2 \leq p \leq 30$.

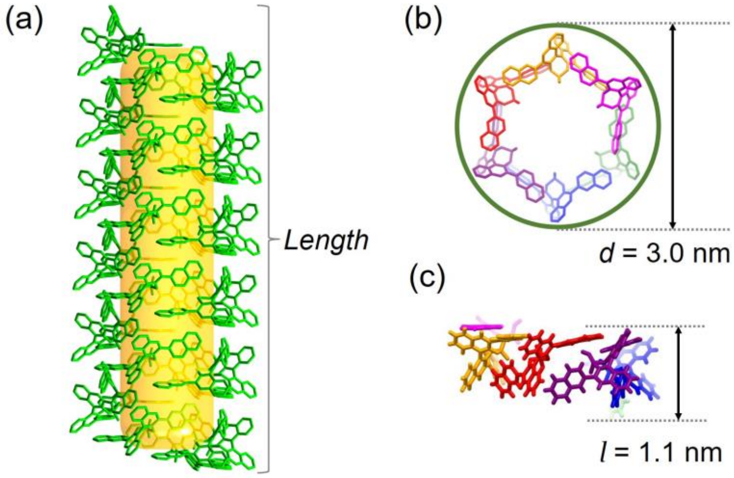

Supplementary Figure 10. The measured parameter of nanotube size from single crystal structure corresponding to the above equation. (a) the axial length as the length of cylinder; (b) the external size of hexagon as the diameter of cylinder; (c) the assembled hexagon as one layer of the nanotube.

In our case, we used the following values to calculate the length of the nanotube: *η* was measured to be 1.90×10^{-3} Pa • s^{-1}, T = 300 K, $k = 1.38 \times 10^{-23}$ $J \cdot K^{-1}$, d = 3.0 nm, the length of the monolayer (hexagon) was measured to be 1.1 nm from crystal data. Thus, applying the modified Stokes-Einstein equation above, we could calculate the diffusion coefficient (*D*)

corresponding to the consecutive stacking layers of assembled hexagon under valid condition (stacking layer from 6 to 30 in relative to $2 \leq p \leq 30$) and plot the curve of diffusion coefficient *versus* stacking layer to find out the real stacking layer of nanotube corresponding to the tested *D* value under variable concentration conditions.

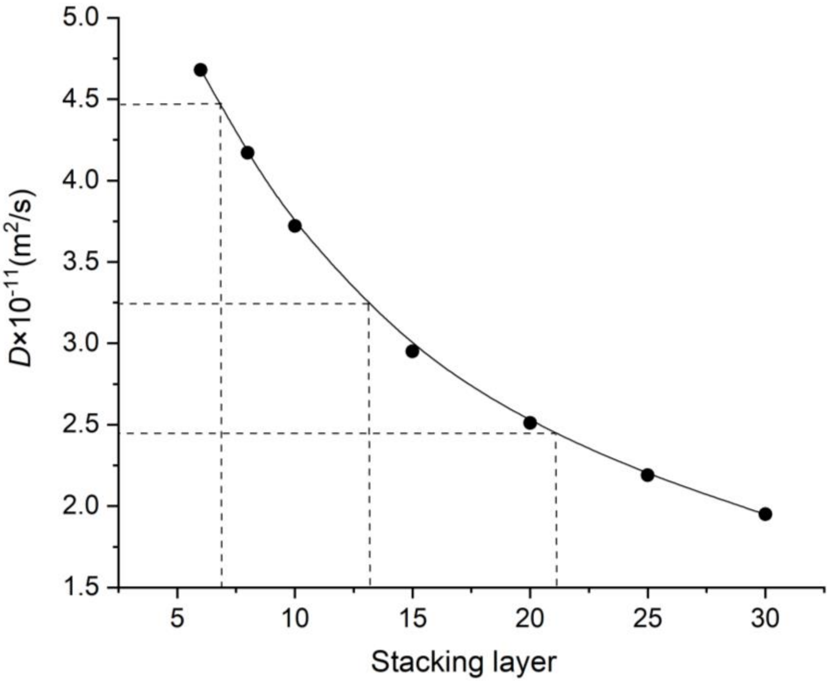

Supplementary Figure 11. The plot of diffusion coefficient *versus* the stacking layer of nanotube calculated by Stokes-Einstein equation with cylinder model approximation (the crosswires give the real stacking layer number corresponding to the tested *D* values under certain concentration conditions).

Supplementary Table 4. The estimated stacking layer number and corresponding length of nanotube based on cylinder model analysis at different concentration.

Conc. (mM)	log*D*	D (m^2/s)	Stacking layer	Length (nm)
8.4	-10.35	4.50×10^{-11}	7	7.7
10.0	-10.49	3.25×10^{-11}	13	14.3
12.0	-10.61	2.44×10^{-11}	21	23.1

The solution becomes viscous and difficult for NMR testing at higher concentration beyond

12.0 mM.

Due to the limitation that the cylinder model is only valid for cylinders with 2≤*p*≤30, alternatively, the bead model shows priority to the cylinder model for short cylinders (*p*<2).[4] In our case, two tested *D* values under relative low concentrations (2.8 mM, 5.6 mM) are both above the valid region calculated by the cylinder model, indicating short cylinders are formed under these conditions. As a result, we used the bead model to treat this situation. The BINOL-phosphate as stacking monomer was approximated by two identical, partially overlapping beads with radius of 4.8 Å for calculation of the hydrodynamic parameters. A series of diffusion coefficients against variable stacking monomer number were calculated for the bead model structures using HI4 program.

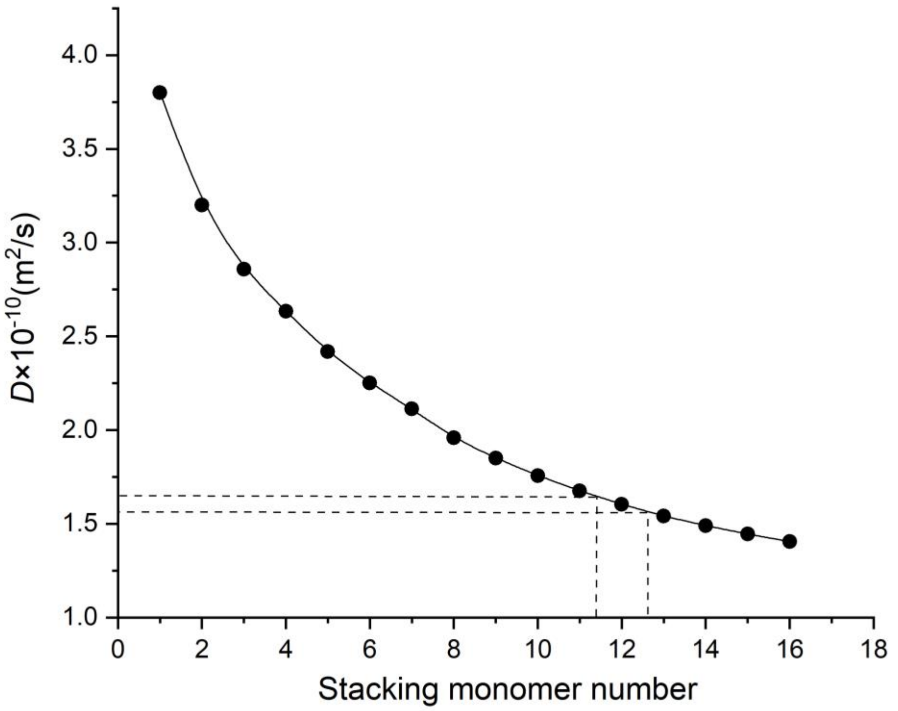

Supplementary Figure 12. The plot of calculated diffusion coefficient *versus* the stacking number of BINOL-phosphate monomer by the bead model (the crosswires give the real stacking number of monomer corresponding to the tested *D* values under relative low concentrations).

Supplementary Table 5. The estimated stacking layer and corresponding length of nanotube based on bead model analysis at different concentrations.

Conc. (mM)	log*D*	D (m^2/s)	Stacking monomer number	Stacking layer[a]	Length (nm)
2.8	-9.78	1.65×10^{-10}	11.5	2	2.2
5.6	-9.81	1.56×10^{-10}	12.6	2	2.2

[a] Because the assembled hexagon as one stacking layer contains six BINOL-phosphate monomers, therefore, the two estimated stacking monomer number around 12 means a short cylinder of two stacking layers was formed under two low concentration conditions.

1.7 TEM testing

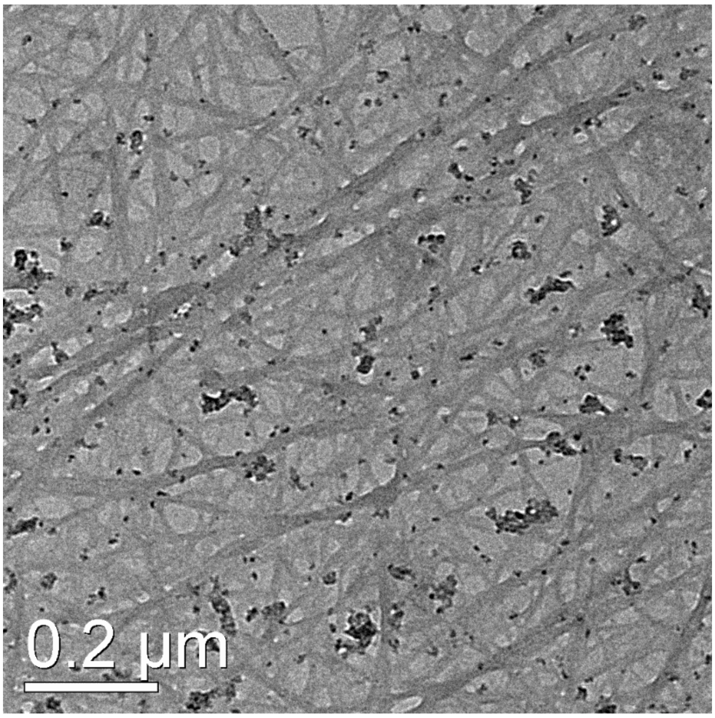

Supplementary Figure 13. The TEM image of assembled nanofibers based on chiral BINOL-phosphate component from aqueous solution.

2. Catalytic Investigation

2.1 Synthesis of substrates

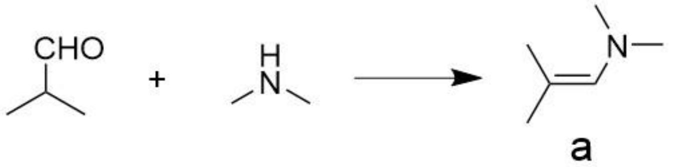

N,N-dimethylisobutenylamine (a)

The procedure was adopted according to the reported literature.[5] Dimethylamine (30% in methanol, 17.6 g, 120 mmol) was slowly added to a stirred solution of isobutyraldehyde (7.2 g, 100 mmol) in diethyl ether (50 mL) at 0 °C, followed by the addition of anhydrous Na_2SO_4 (16 g). The mixture was stirred vigorously for 20 min and transferred to another flask, another batch of anhydrous Na_2SO_4 (8 g) was added at 0 °C and the mixture was continuously stirred for 10 min. Again, the solution was transferred to another flask, anhydrous Na_2SO_4 (5 g) was added and the mixture was stirred for 10 min at 0 °C. After removal of anhydrous Na_2SO_4, 4 Å molecular sieve (9 g) was added and the mixture was stirred slowly at room temperature for 5 h. Finally, the resulting solution was subjected to distillation. The fraction of around 85 °C was collected and the desired enamine product was obtained in 15% yield (1.5 g). ^{1}H NMR (400 MHz, $CDCl_3$): δ 5.32 (m, 1H), 2.38 (s, 6H), 1.68 (s, 3H), 1.60 (s, 3H).

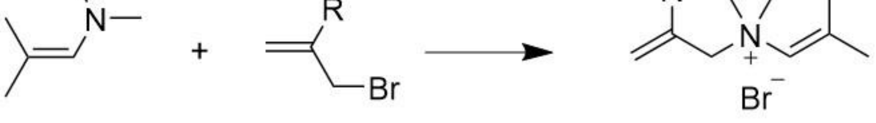

R: H (S1)
R: Me (S2)

N-allyl-*N,N*,2-trimethylprop-1-en-1-aminium (S1)

The procedure was used according to the reported literature.[6] To a stirred solution of *N,N*-dimethylisobutenylamine (0.7 g, 7.1 mmol) in acetonitrile (10 mL) was dropwise added 3-

bromoprop-1-ene (672 μL, 7.8 mmol) in acetonitrile (10 mL) at 0 °C.The reaction mixture was stirred at 0 °C for 48 h. The solvent was removed under reduced pressure. The resulting raw product was washed with dry diethyl ether for three times (20 mL×3). Finally, the pure oily product was obtained in 80% yield (1.2 g). ^{1}H NMR (400 MHz, $CDCl_3$): *δ* 5.98 (s, br, 1H), 5.88 (m, 1H), 5.78 (s, 1H), 5.70 (m, 1H), 4.59 (d, *J* = 6.8 Hz, 2H), 3.62 (s, 6H), 2.06 (d, *J* = 1.2 Hz, 3H), 1.88 (d, *J* = 1.2 Hz, 3H).

_N,N_,2-trimethyl-_N_-(2-methylallyl)prop-1-en-1-aminium (S2)

The procedure was similar to that of S1, except that the reactant 3-bromoprop-1-ene was replaced by 3-bromo-2-methylprop-1-ene (783 μL, 7.8 mmol). The pure oily product was obtained in 75% yield (1.3 g). ^{1}H NMR (400 MHz, $CDCl_3$): *δ* 5.97 (s, br, 1H), 5.48 (s, 1H), 4.38 (s, 2H), 3.64 (s, 6H), 2.03 (m, 3H), 1.93 (s, 3H), 1.87 (s, 3H).

2.2 ITC titration between substrates and nanotube

(a)

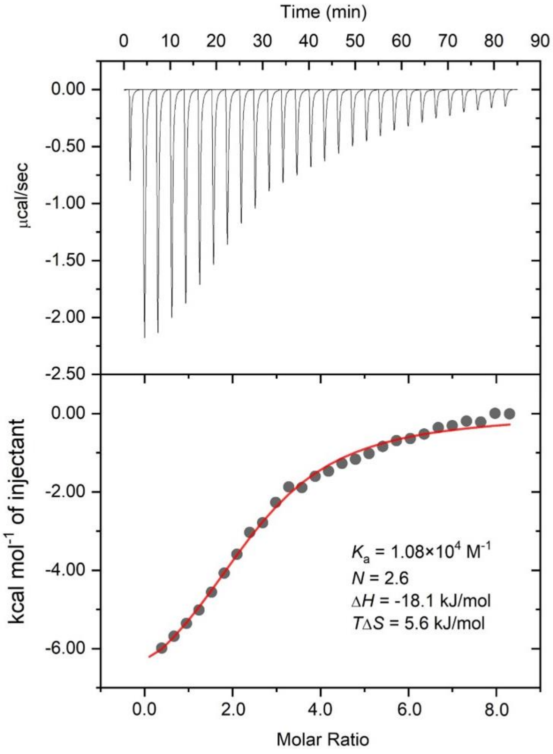

(b)

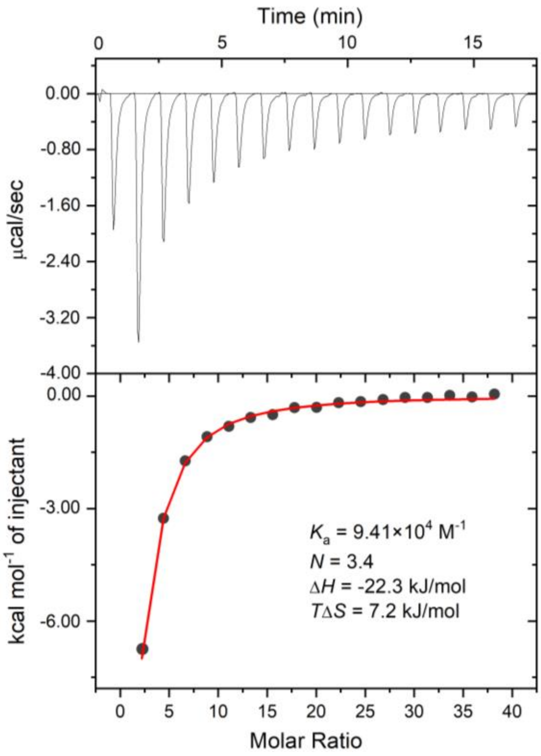

Supplementary Figure 14. ITC titration plots (the molar ratio refers to the substrates as guest over assembled hexagon as host) of **S1** (a) and **S2** (b) to nanotube in aqueous solution at 298 K. (*Note*: the conversion of S1 and S2 are below 8% and 10% under the main course of titration process)

2.3 General procedure for catalytic study

The substrates (2.8 mM) were introduced to the solvent system (600 μL, DMSO-d_6 or DMSO-d_6/D_2O mixture) containing specific catalysts (5.6 mM). The solutions were adjusted to pD = 8.0 and transferred to NMR tube. The reaction mixture was placed at the set temperature and monitored by in situ NMR spectroscopy to obtain the corresponding kinetic data.

2.4 Reaction rate constant fitting and control experiment

Although the whole chemical transformation includes two steps, however, the second step of hydrolysis is largely faster than the first step of intramolecular rearrangement, thus the first step is the rate-determining step and the first-order reaction law could be applied for kinetic analysis.[7]

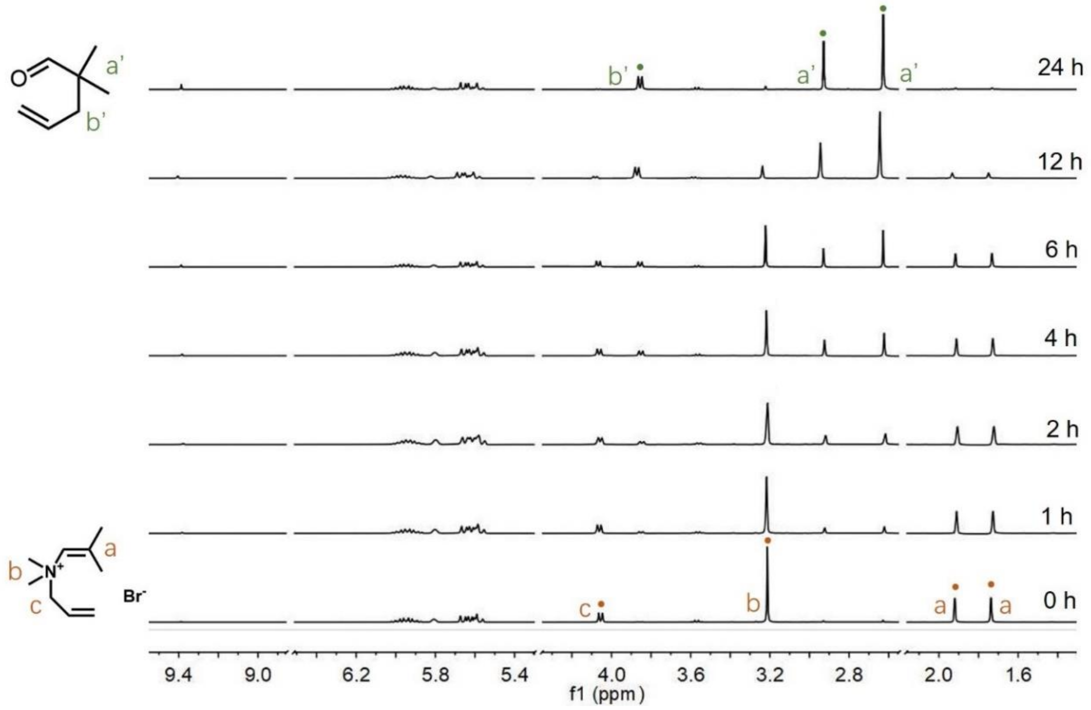

Supplementary Figure 15. In situ ^{1}H NMR spectra monitoring the 3-aza-Cope rearrangement of substrate 1 (**S1**) at 50 °C without catalyst. (400 MHz, DMSO-d_6:D_2O = 1:2)

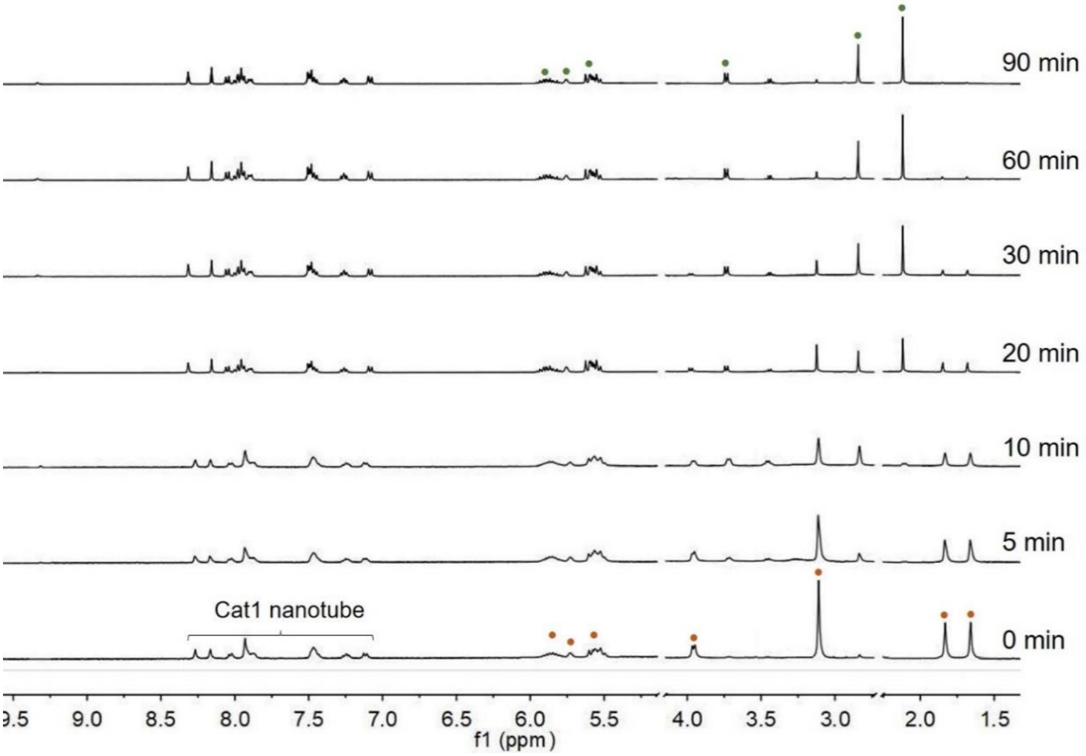

Supplementary Figure 16. In situ ^{1}H NMR spectra monitoring the 3-aza-Cope rearrangement of substrate 1 (**S1**) at 50 °C with Cat1 nanotube catalyst (orange dots represent the substrate, green dots represent the product). (400 MHz, DMSO-d_6:D_2O = 1:2)

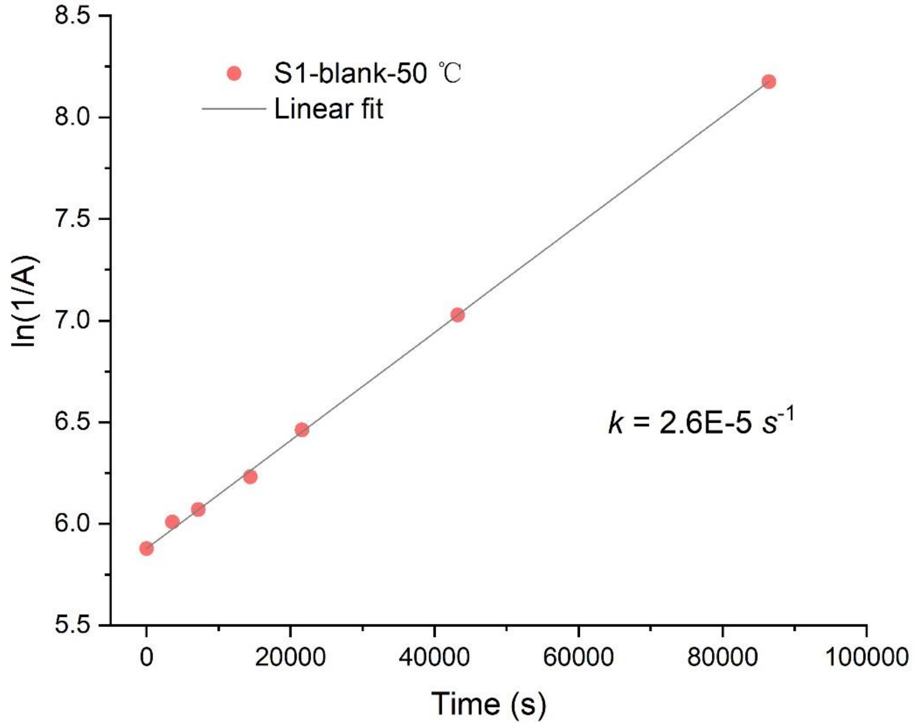

Supplementary Figure 17. Kinetic fitting of **S1** reaction without catalyst at 50 ℃ with the first-order reaction rate equation.

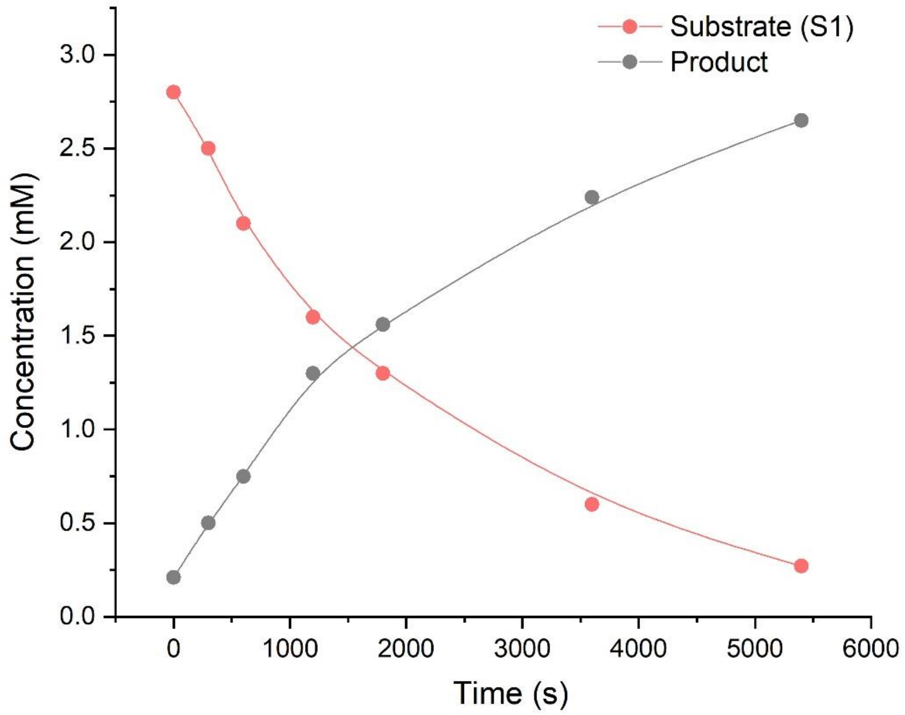

Supplementary Figure 18. The concentration of **S1** and corresponding product along with reaction time under the Cat1(nanotube) catalyst at 50 ℃.

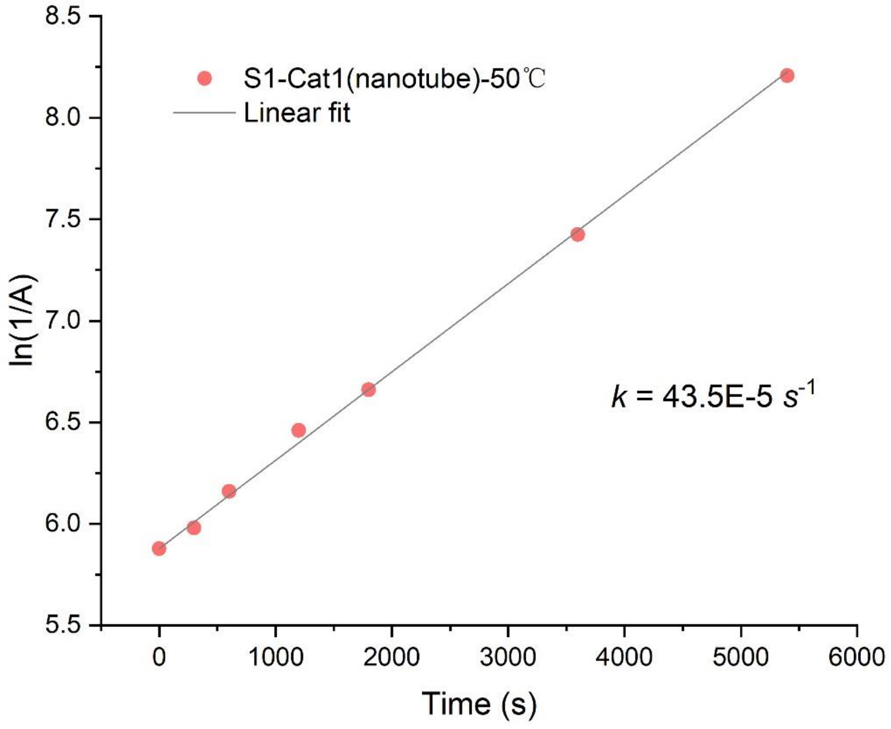

Supplementary Figure 19. Kinetic fitting of **S1** reaction under Cat1(nanotube) catalyst condition at 50 °C with the first-order reaction rate equation.

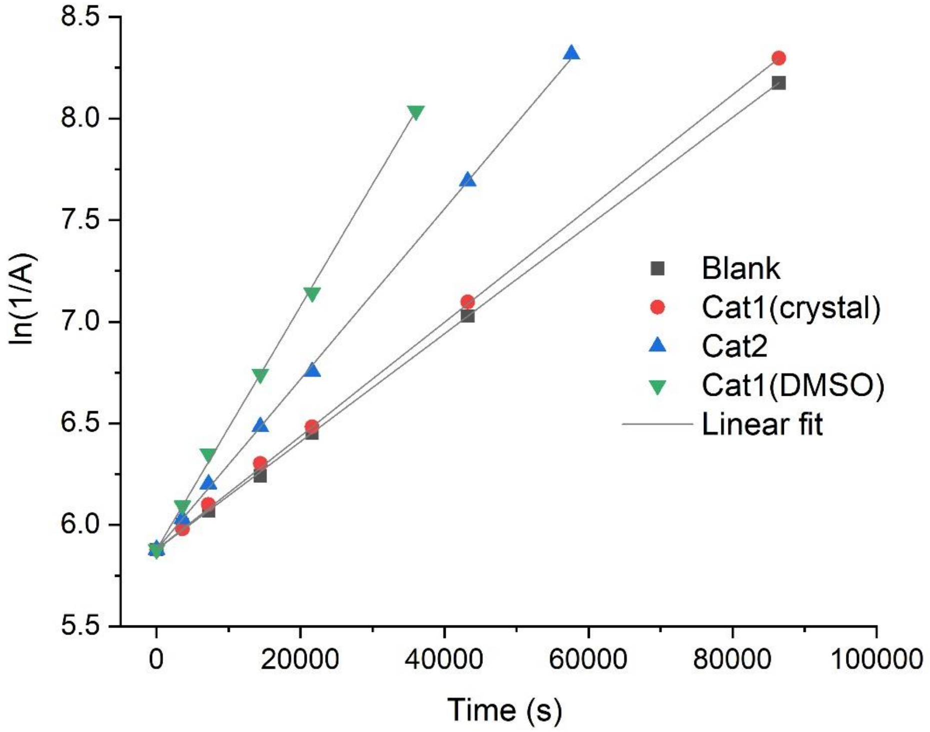

Supplementary Figure 20. Comparison of kinetic fitting the control experimental data of **S1** reaction at 50 °C with the first-order reaction rate equation.

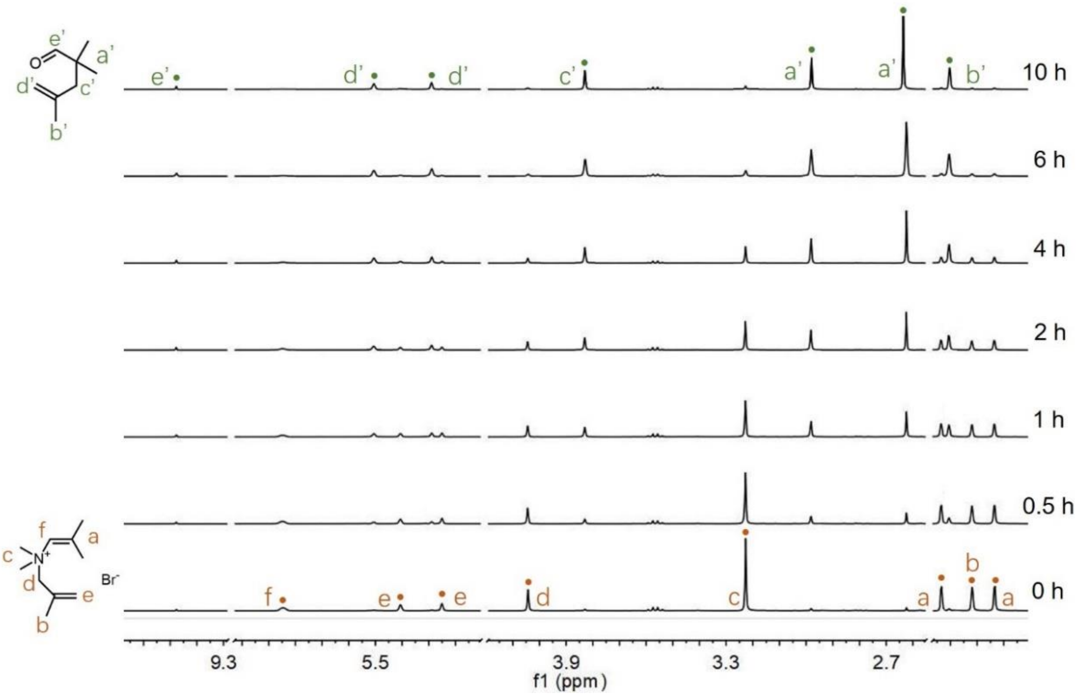

Supplementary Figure 21. In situ ^{1}H NMR spectra monitoring the 3-aza-Cope rearrangement of substrate 2 (**S2**) at 50 °C without catalyst. (400 MHz, DMSO-d_6:D_2O = 1:2)

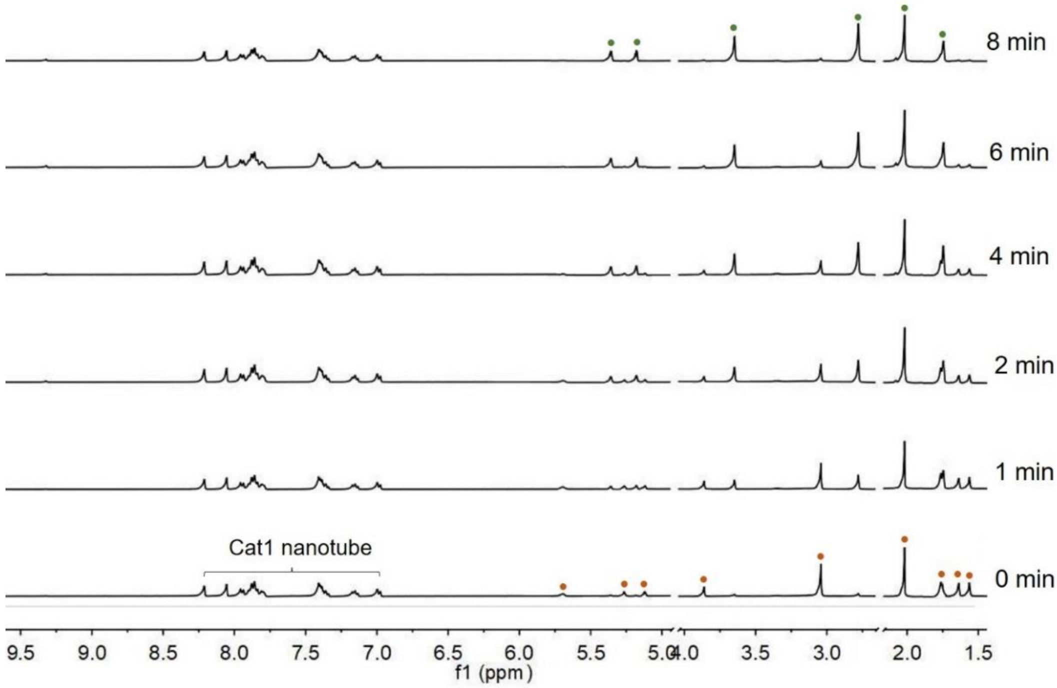

Supplementary Figure 22. In situ ^{1}H NMR spectra monitoring the 3-aza-Cope rearrangement of substrate 2 (**S2**) at 50 °C with Cat1 nanotube catalyst (orange dots represent the substrate, green dots represent the product). (400 MHz, DMSO-d_6:D_2O = 1:2)

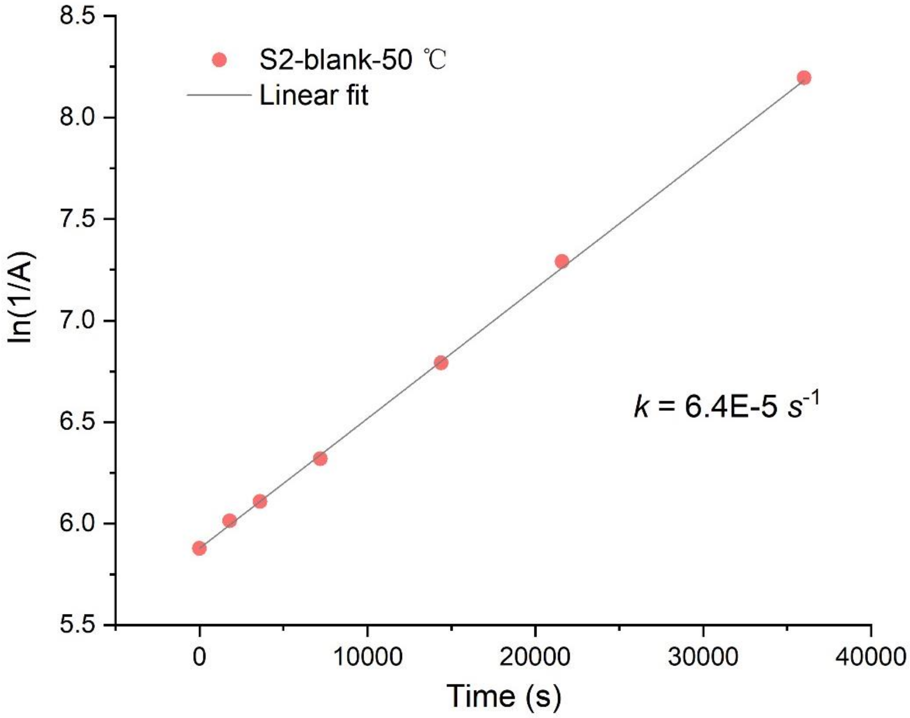

Supplementary Figure 23. Kinetic fitting of **S2** reaction without catalyst at 50 °C with the first-order reaction rate equation.

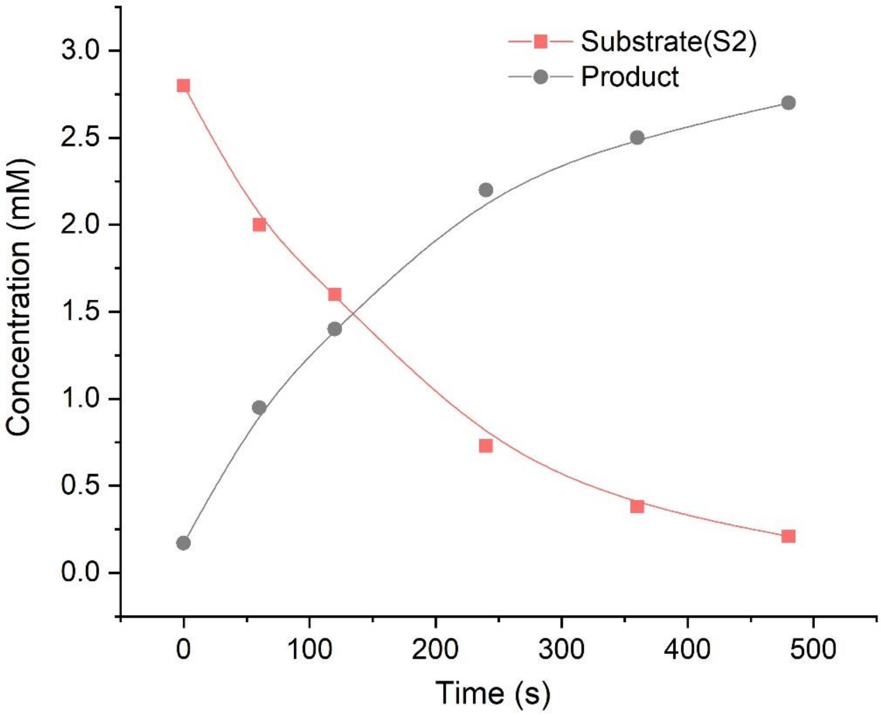

Supplementary Figure 24. The concentration of **S2** and corresponding product along with reaction time under the Cat1(nanotube) catalyst at 50 °C.

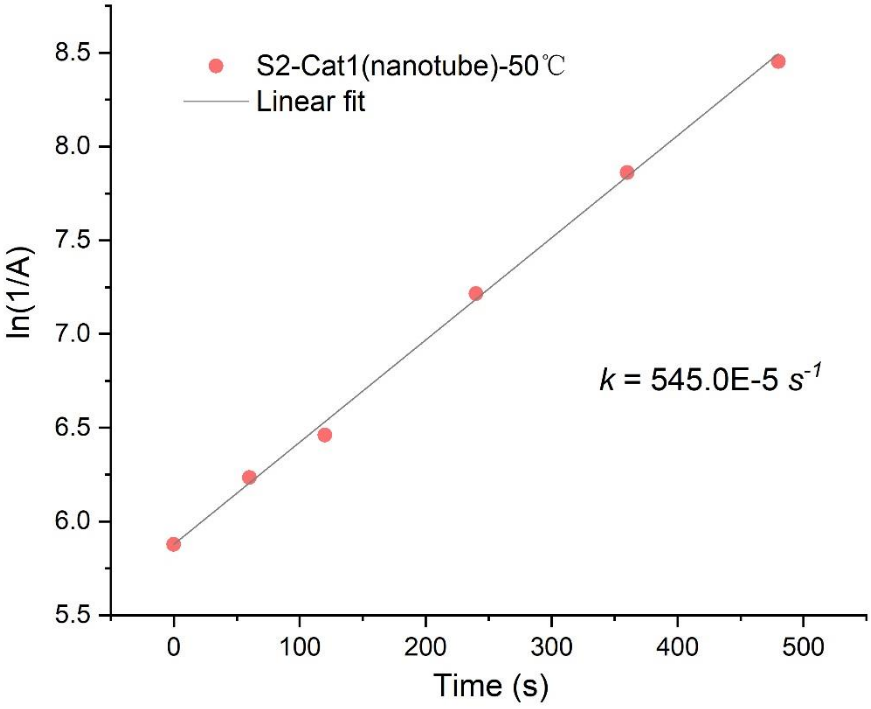

Supplementary Figure 25. Kinetic fitting of **S2** reaction under Cat1(nanotube) catalyst condition at 50 °C with the first-order reaction rate equation.

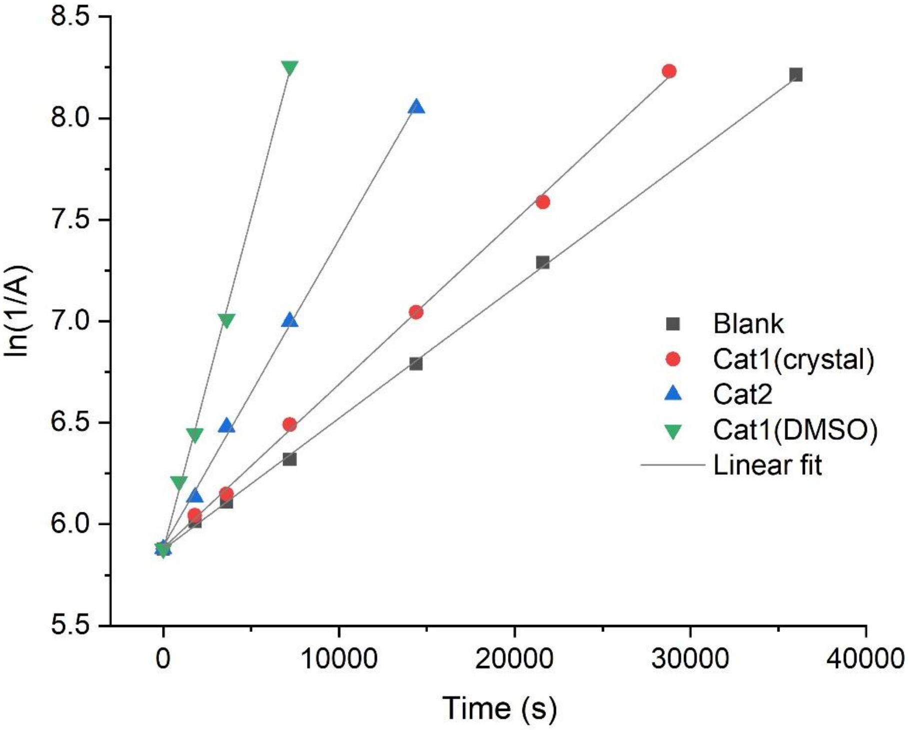

Supplementary Figure 26. Comparison of kinetic fitting the control experimental data of **S2** reaction at 50 °C with the first-order reaction rate equation.

2.5 Enthalpy of activation ($\Delta H^{\ddagger}$) and Entropy of activation ($\Delta S^{\ddagger}$) fitting

In transition state theory, the mathematical expression of Eyring equation is:[8]

$$k = \frac{k_B T}{h} e^{-\frac{\Delta H^{\ddagger}}{RT}} e^{\frac{\Delta S^{\ddagger}}{R}}$$

The linear form of this equation is given below:

$$ln\frac{k}{T} = \frac{-\Delta H^{\ddagger}}{R}\frac{1}{T} + ln\frac{k_B}{h} + \frac{\Delta S^{\ddagger}}{R}$$ (Supplementary Equation 2)

where:

k is the reaction rate constant,

T is the absolute temperature in Kelvin (K),

$\Delta H^{\ddagger}$ is the enthalpy of activation,

R is the gas constant (8.3145 J/mol·K),

k_B is the Boltzmann's constant (1.381 x 10^{-23} J/K),

h is Planck's constant (6.626 x 10^{-34} J·s),

$\Delta S^{\ddagger}$ is the entropy of activation.

The values for $\Delta H^{\ddagger}$ and $\Delta S^{\ddagger}$ can be determined from kinetic data obtained from a $ln\frac{k}{T}$ versus $\frac{1}{T}$ plot. The Equation is a straight line with negative slope, $\frac{-\Delta H^{\ddagger}}{R}$, and a y-intercept, $ln\frac{k_B}{h} + \frac{\Delta S^{\ddagger}}{R}$.

(a)

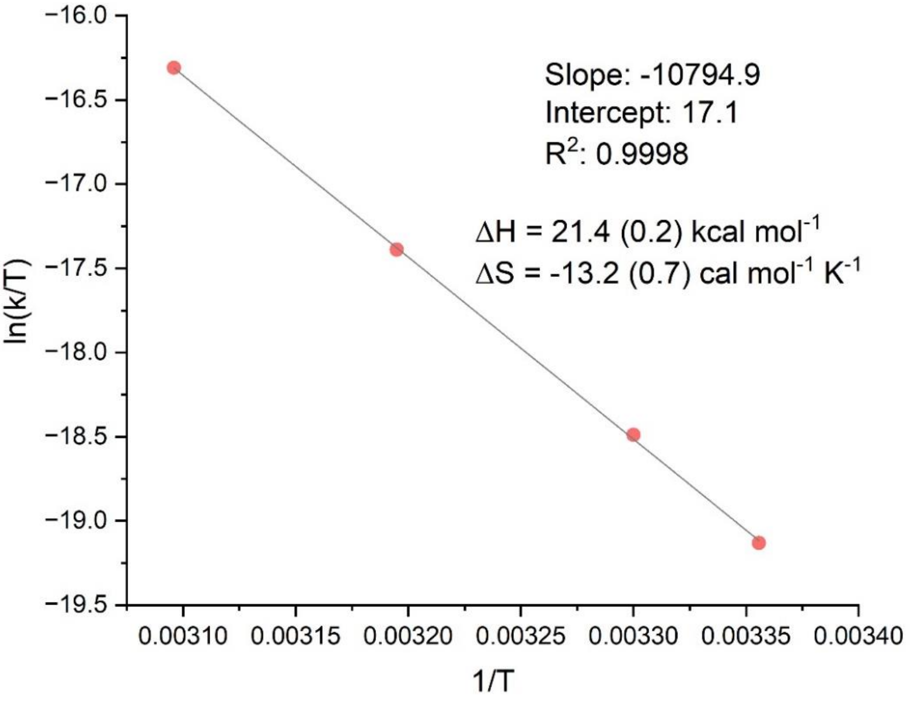

(b)

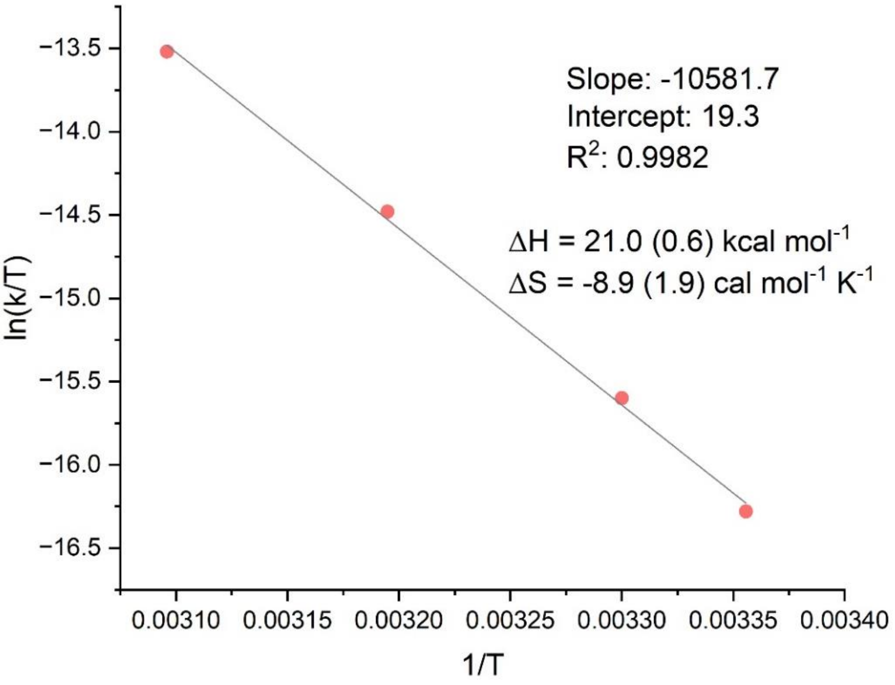

Supplementary Figure 27. Eyring equation fitting $\Delta H^{\ddagger}$ and $\Delta S^{\ddagger}$ for **S1** rearrangement under free (a) and Cat1 nanotube (b) catalytic conditions. (Temperature setpoint: 25 °C, 35 °C, 45 °C, 50 °C)

(a)

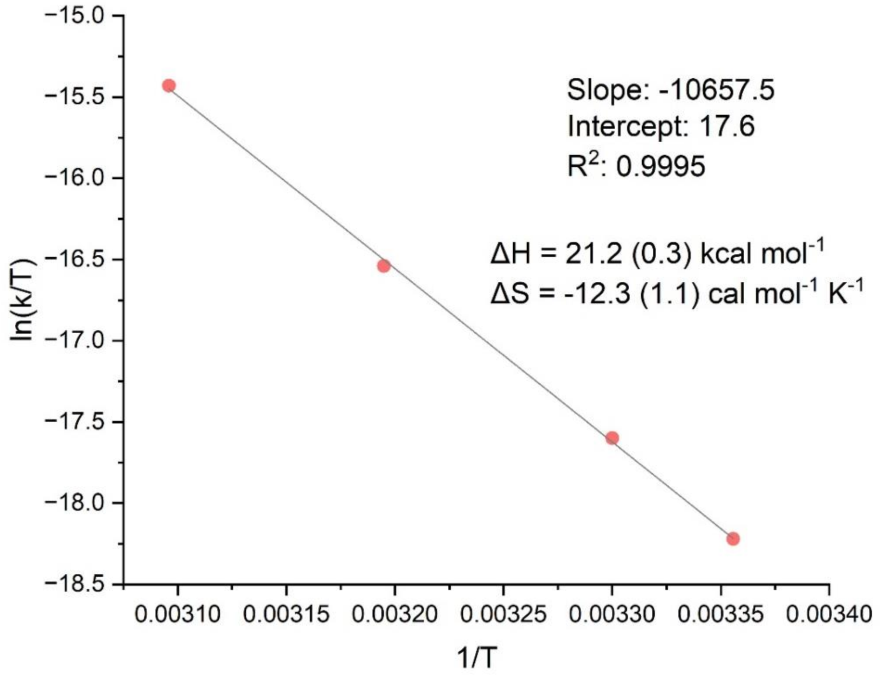

(b)

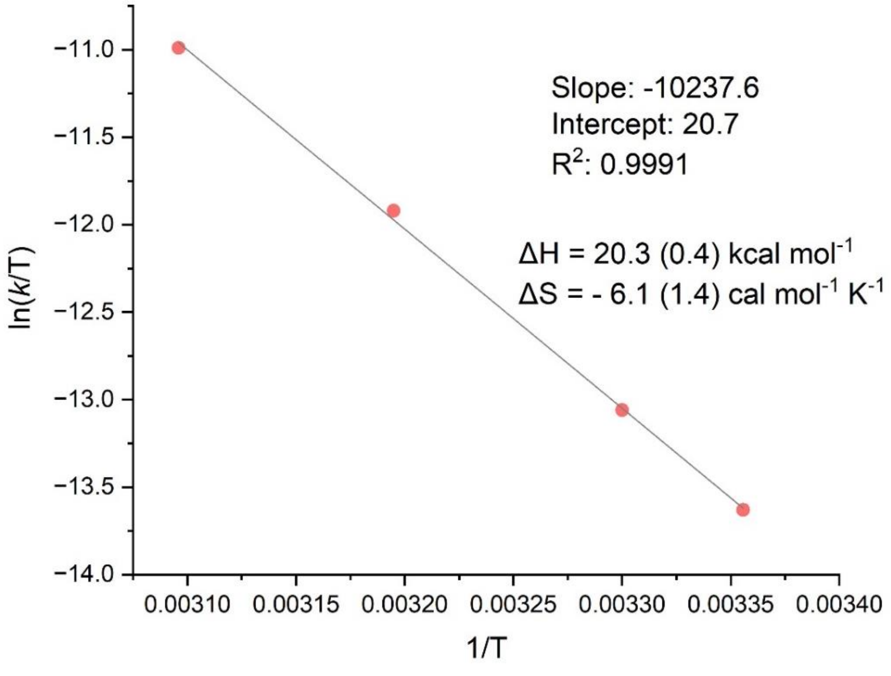

Supplementary Figure 28. Eyring equation fitting $\Delta H^{\ddagger}$ and $\Delta S^{\ddagger}$ for **S2** rearrangement under free (a) and Cat1 nanotube (b) catalytic conditions. (Temperature setpoint: 25 °C, 35 °C, 45 °C, 50 °C)

2.6 NOESY measurement

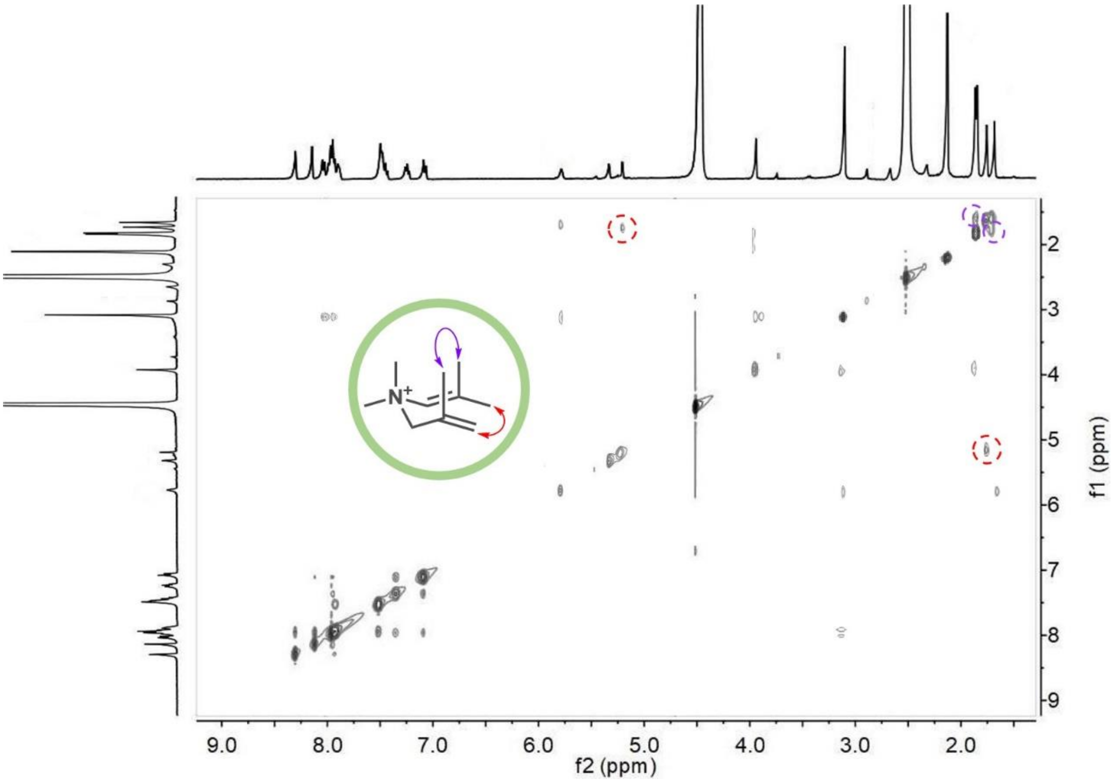

Supplementary Figure 29. ^{1}H-^{1}H NOESY spectrum of **Cat1** aqueous solution containing **S2** as guest; inserted is the proposed self-folded conformation of **S2** in the confined space of nanotube, the circled cross-peaks are assigned to the intramolecular long-distance correlations. (400 MHz, 298 K, DMSO-d_6 : D_2O = 1:2)

2.7 Asymmetric catalysis investigation

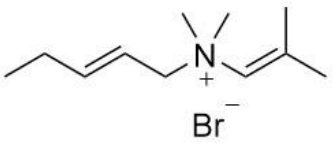

(*E*)-N,N-dimethyl-N-(2-methylprop-1-en-1-yl)pent-2-en-1-aminium bromide (S3)

The procedure was similar to that of S1, except that the reactant 3-bromoprop-1-ene was replaced by (*E*)-1-bromopent-2-ene. The product was isolated as a colorless oil in 80% yield. ^{1}H NMR (400 MHz, $CDCl_3$): δ 6.22 (d, J = 14.8 Hz, 1H), 5.90 (s, br, 1H), 5.41 (d, J = 15.0 Hz, 1H), 4.42 (d, J = 7.2 Hz, 2H), 3.45 (s, 6H), 2.05 (m, 2H), 1.85 (d, J = 1.5 Hz, 3H), 1.75 (d, J = 1.5 Hz, 3H), 0.83 (m, 3H).

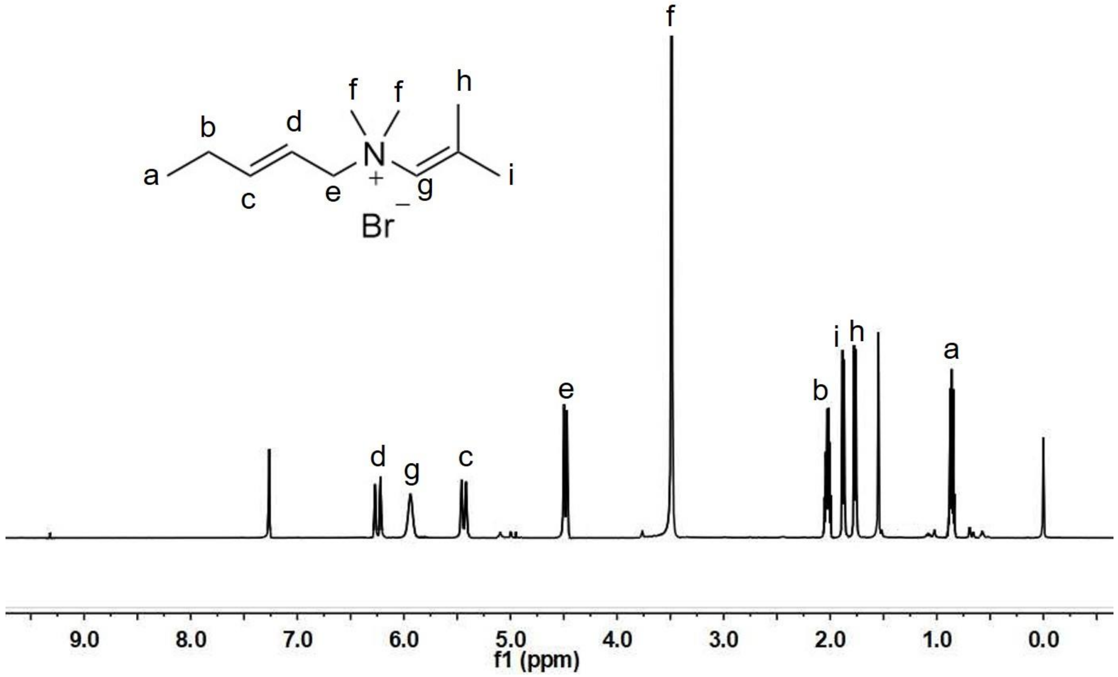

Supplementary Figure 30. ^{1}H NMR spectrum of **S3** (400 MHz, $CDCl_3$, 298 K).

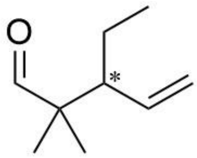

3-ethyl-2,2-dimethylpent-4-enal (chiral-potential product)

The **S3** as substrate (0.02 mmol) was introduced to the DMSO/H_2O mixture (200 μL + 400 μL) containing R or S-nanotube as catalyst (5.6 mM, based on phosphate monomer). The reaction solution was stirred at room temperature for 1d and extracted by $CDCl_3$ (500 μL). The obtained product was used for the following ^{1}H NMR and chiral GC analysis (the temperature gradient was set to begin at 60 °C with an increase of 0.25 °C/min).

^{1}H NMR (400 MHz, $CDCl_3$): δ 9.32 (s, 1H), 5.35 (d, *J* = 15.0 Hz, 1H), 5.01 (d, *J* = 10.0 Hz, 1H), 4.81 (d, *J* = 15.0, 1H), 1.75 (m, 1H), 1.10 (m, 1H), 0.89 (m, 1H), 0.80 (s, 3H), 0.65 (s, 3H), 0.61 (t, *J* = 6.0 Hz, 3H).

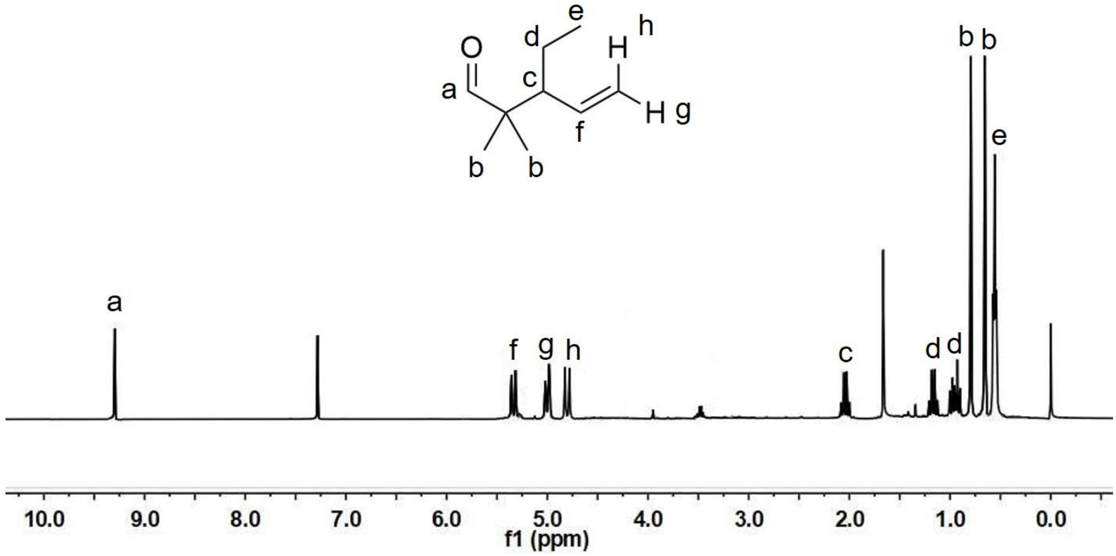

Supplementary Figure 31. ^{1}H NMR spectrum of catalytic product corresponding to **S3** (400 MHz, $CDCl_3$, 298 K).

(a)

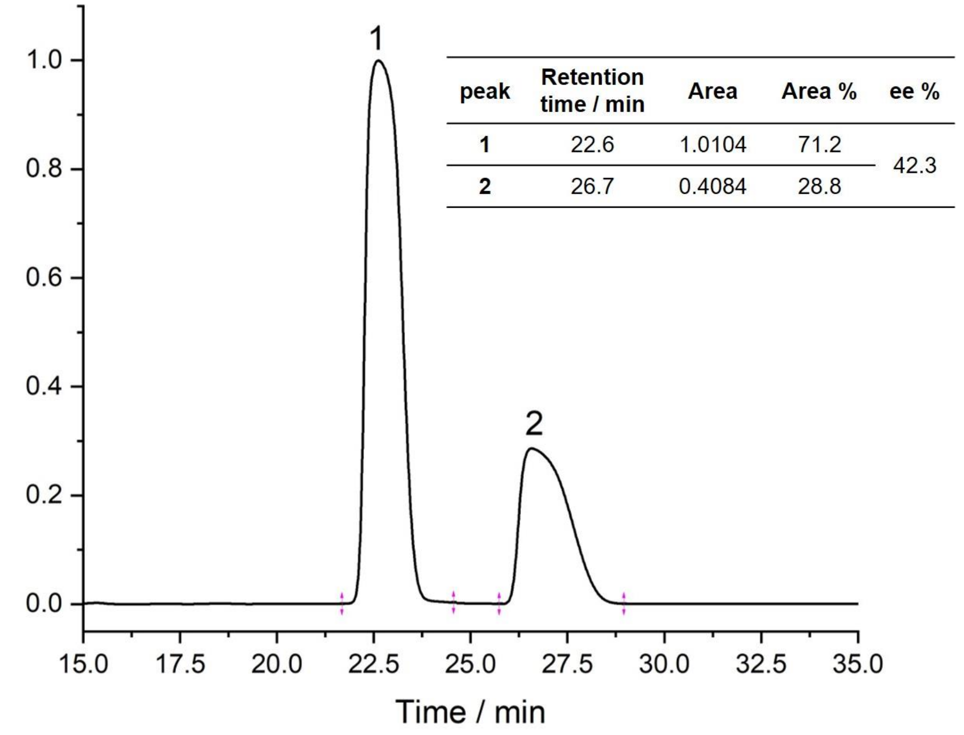

peak	Retention time / min	Area	Area %	ee %
1	22.6	1.0104	71.2	42.3
2	26.7	0.4084	28.8	

(b)

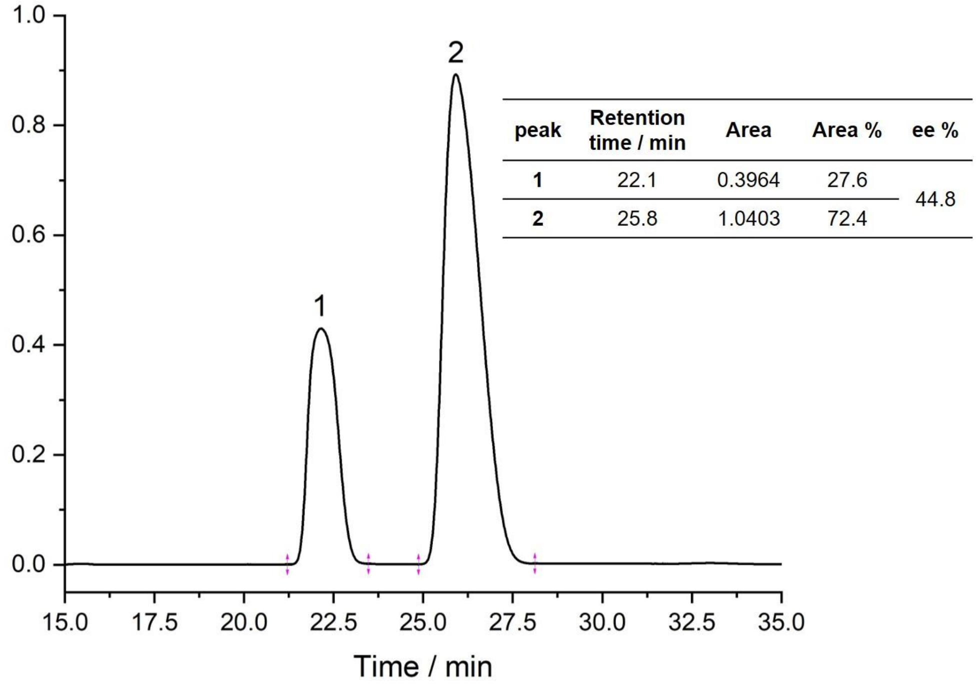

peak	Retention time / min	Area	Area %	ee %
1	22.1	0.3964	27.6	44.8
2	25.8	1.0403	72.4	

Supplementary Figure 32. Selected chiral GC spectrum of catalytic product of **S3** using **R-nanotube** (a) or **S-nanotube** (b) as catalyst. (Inserted tables are the statistical results of two main peaks for enantiomers)

3. Supplementary References

1. Dolomanov, O. V., Bourhis, L. J., Gildea, R. J., Howard, J. A. K. & Puschmann, H. OLEX2: a complete structure solution, refinement and analysis program. *J. Appl. Crystallogr.* **42**, 339-341 (2009).
2. Sheldrick, G. M. SHELXT - integrated space-group and crystal-structure determination. *Acta Crystallogr. A Found. Adv.* **71**, 3-8 (2015).
3. Bourhis, L. J., Dolomanov, O. V., Gildea, R. J., Howard, J. A. & Puschmann, H. The anatomy of a comprehensive constrained, restrained refinement program for the modern computing environment - Olex2 dissected. *Acta Crystallogr. A Found. Adv.* **71**, 59-75 (2015).
4. Wong, A., Ida, R., Spindler, L. & Wu, G. Disodium guanosine 5'-monophosphate self-associates into nanoscale cylinders at pH 8: a combined diffusion NMR spectroscopy and dynamic light scattering study. *J. Am. Chem. Soc.* **127**, 6990-6998 (2005).
5. Chen, X., Fan, H., Zhang, S., Yu, C. & Wang, W. Facile installation of 2-reverse prenyl functionality into indoles by a tandem N-alkylation-aza-cope rearrangement reaction and its application in synthesis. *Chem. Eur. J.* **22**, 716-723 (2016).
6. Fiedler, D., van Halbeek, H., Bergman, R. G. & Raymond, K. N. Supramolecular catalysis of unimolecular rearrangements: substrate scope and mechanistic insights. *J. Am. Chem. Soc.* **128**, 10240-10252 (2006).
7. Fiedler, D., Bergman, R. G. & Raymond, K. N. Supramolecular catalysis of a unimolecular transformation: aza-cope rearrangement within a self-assembled host. *Angew. Chem. Int. Ed.* **43**, 6748-6751 (2004).
8. Espenson, J. H. *Chemical kinetics and reaction mechanisms.* (McGraw-Hill, 1981).
